# Artificial embryonic node elucidates the role of flow in left-right symmetry breaking in vertebrates

**DOI:** 10.1126/sciadv.aec2328

**Published:** 2026-03-25

**Authors:** Tanveer ul Islam, Ishu Aggarwal, Yves Bellouard, Patrick R. Onck, Jaap M. J. den Toonder

**Affiliations:** ^1^ Microsystems Section, Mechanical Engineering, Eindhoven University of Technology, Eindhoven 5612 AZ, Netherlands.; ^2^ Institute for Complex Molecular Systems, Eindhoven University of Technology, Eindhoven 5612 AZ, Netherlands.; ^3^ Galatea Lab, STI/IEM, Ecole polytechnique fédérale de Lausanne (EPFL), CH-2002 Neuchâtel 2, Switzerland.; ^4^ Zernike Institute for Advanced Materials, University of Groningen, Groningen 9747 AG, Netherlands.

## Abstract

During the embryonic development of vertebrates, initially, symmetric embryos develop asymmetrically arranged organs. The asymmetry initiates with the formation of a small fluid-filled cavity on the embryo called the embryonic node, which contains motile cilia that generate specific flow patterns. The mechanism by which this nodal flow is sensed and causes asymmetry development has remained elusive despite major experimental and computational efforts. Existing hypotheses focus on either mechanical or chemical signaling processes. We report an experimental artificial embryonic node that, combined with numerical simulations, enables in-depth investigation of nodal flow and its role in left-right asymmetry development. Dissimilar fluid velocity profiles develop around primary cilia on the left and right nodal sides, producing distinct cilium bending. Also, the distribution of signaling particles with specific diffusivities exhibits spatial and temporal asymmetry. Together, our results support both mechanical and chemical sensing hypotheses and suggest a potential synergy between the two sensing mechanisms for the enhanced robustness of left-right asymmetry development.

## Teaser

Investigation of nodal flow in artificial embryonic node reveals the mechanism behind left-right symmetry breaking in vertebrates.

## INTRODUCTION

**Fig. 1.**
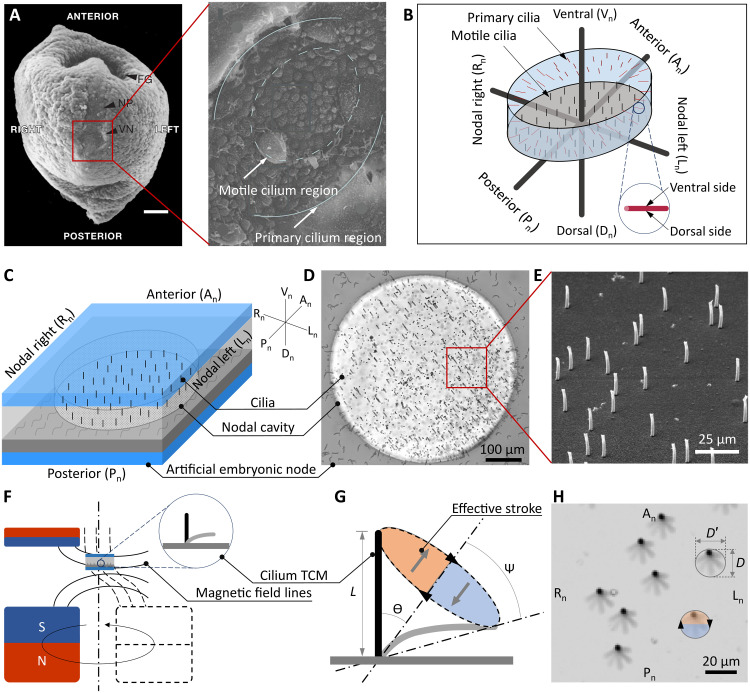
Artificial embryonic node fabrication and actuation. (**A**) Mouse embryo at 7.5 days postcoitum, including the embryonic node (red square box); scale bar: 100 μm (*42*). A magnified view of the embryonic node shows the region of motile cilia (inner dashed circle) and the region of immotile/primary cilia between the inner and outer circles (*43*). (**B**) Schematic representation of the embryonic node. Following the convention in this field, the node is seen from the posterior side, and thus, the left side of the node is on the viewer’s right. A zoomed-up view of a primary cilium shows its dorsal and ventral sides relative to the node. The primary cilia are attached to the vertical node walls and oriented perpendicularly to the motile cilia. (**C**) Schematic of a circular artificial embryonic node containing, from bottom to top, a glass substrate, a PDMS layer supporting the nanomagnetic artificial cilia, the nodal cavity, and a top glass plate. The nodal cavity is filled with a Newtonian fluid containing tracer particles. Labels and axes in the top right represent the nodal axes relative to the embryo. (**D**) Bright-field image of one of our circular artificial embryonic nodes showing cilia and tracer particles inside the nodal cavity. (**E**) Scanning electron microscopy image of artificial cilia made after critical point drying (radius, 1 μm; length, 23 μm). (**F**) Schematic of the two-magnet actuation arrangement and the field lines for two extreme positions of the rotating magnet, relative to the artificial embryonic node, and a zoomed-up view of a cilium under the two extreme magnetic field orientations. (**G**) Schematic of a TCM of a single cilium indicating forward/effective and backward strokes. (**H**) Superimposed top-view snapshots of cilia undergoing TCM. The major and minor axes of the ellipse traced by the tip are represented by *D* and *D*′, respectively.

Humans, like most other vertebrates, appear externally left-right symmetric but show asymmetry in the placement of their internal organs: For example, the heart and stomach are positioned toward the left and the liver toward the right of the body (*1*, *2*). The development of this left-right asymmetry in vertebrates has been traced back to an elusive process during the early stages of embryonic development, happening in a small cavity that contains cilia (*3*). Mouse embryos, which have been extensively studied, develop a closed triangular cavity on their ventral side, 10 to 20 μm deep and 50 to 100 μm across, which is called the embryonic node or left-right organizer [see Fig. 1 (A and B)] (*3*, *4*). The node shape is different for different vertebrates, for example, circular in rabbit and rectangular in medaka fish (*5*). The node is filled with an extraembryonic fluid, and it is closed on the top side by a membrane called Reichert’s membrane, whereas the bottom surface is lined with a layer of a few hundred monociliated cells (*4*, *6*). The left-right symmetry breaking process commences with the degradation of a gene called *Dand5* (*7*), also known as *Cerl2 or Cer2* (*8*), followed by a signaling cascade, i.e., the sequential activation/expression of genes called *nodal*, (*9*) *lefty 1,2* (*10*), and *Pitx2* (*11*, *12*) in the cells present inside and on the left side of the node (*13*). The cilia within the node have an average length and diameter of 5 and 0.3 μm, respectively (*4*). They have a 9 + 0 axoneme configuration, initially thought to be immotile (*14*) but later proven to perform a rotary motion at a frequency of around 10 Hz, producing a net flow of the extraembryonic fluid, which is referred to as nodal flow (*3*). The nodal flow development precedes the signaling cascade event described above and was first hypothesized and demonstrated through simulations (*15*) to occur because of a tilted rotation of the cilia, known as tilted conical motion (TCM). During each rotation cycle, the cilia exhibit an effective (or forward) stroke to the nodal left moving perpendicularly aligned to the surface during which their effect on the fluid is maximized and a recovery (or backward) stroke to the nodal right moving close to the surface during which their effect is minimal, reaching extreme bending angles of around 90° (*5*). The motion globally produces a net leftward flow near the bottom ciliated surface along with a returning rightward flow in the upper part of the node (*5*). It has been hypothesized that the nodal flow triggers the signaling cascade, but determining the exact way in which this happens has been challenging, partly due to the continuous spatiotemporal interactions within the node and the difficulty to visualize these interactions and the nodal flow in the very small node, which lives for only a short time period of just a few hours (*16*, *17*). On the basis of previous research, the symmetry breaking process has been hypothesized to happen because of one of the following three mechanisms: (i) generation of a flow-driven morphogen concentration gradient, which is sensed in the node (chemosensing) (*5*); (ii) transportation by the flow of nodal vesicular parcels (NVPs) containing signaling molecules to one side of the node and their rupture there to trigger the signaling cascade (chemosensing) (*18*); and (iii) detection of flow by immotile/primary cilia, present along the outer region (crown) of the node (see Fig. 1A), that bend because of the flow generated by motile cilia in the central region of the node (mechanosensing), which is known as the two-cilium hypothesis (*19*, *20*).

Numerical simulations of the nodal flow and experimental analysis of the node have been carried out to test the three hypotheses. Different approaches have been adopted. The cilia have been represented in simulations by discrete tilted vortices, called rotlets, which were found to produce a leftward flow in the central region and a returning flow along the upper and lower surfaces of the node (*15*). By incorporating a fluid-structure (fluid-cilia) interaction model (*21*) in a three-dimensional closed space with symmetrically placed cilia, a vortical flow in the lower part and a rightward directional flow in the upper part were predicted (*22*, *23*), but these flow patterns have lacked experimental verification. In addition to evaluating the nodal flow, simulations have shown that morphogens can develop a stable short-time gradient if they undergo inactivation on a very short timescale (a maximum of less than 5 s), but this has not been observed experimentally up to now (*15*, *24*). In experiments with an externally imposed artificial flow, typical left-right symmetry breaking was observed even though no morphogen gradient could develop in the open node where the fluid was continuously refreshed (*25*). These results have led to the suggestion that the symmetry breaking process is likely not dependent on the development of a morphogen gradient (*24*). The drawbacks of the morphogen hypothesis are partly evaded by the NVP hypothesis by having the morphogens safely protected in NVPs, which would release their content by rupturing after being driven to the left side of the node (*18*). A meshwork of fibrous strands present on the left half of the bottom nodal surface has been suggested to guide the NVPs toward the left-side crown cells under the leftward nodal flow (*26*). The docking proteins between the guiding fibrous meshwork and the NVPs and the molecular events that would result in the sensing of the NVPs by the left-side crown cells are not known yet and need to be investigated to confirm whether NVPs play any role in the asymmetry development at all (*26*). Unlike the two chemosensing hypotheses, the two-cilium hypothesis relies on the affirmation that immotile cilia are mechanosensory, i.e., they are responsive to calcium ions (Ca^2+^) when deflected, and they respond asymmetrically on the two sides of the node to kick off the asymmetry. The mechanosensory nature was earlier contradictorily found false (*27*) as well as true (*28*) for the nodal cell cilia, but recent investigations have confirmed their sensory nature by directly applying mechanical stimuli through the use of optical tweezers and measuring their response to calcium ions (Ca^2+^) (*29*–*31*). Further analysis revealed the presence of a mechanosensitive cation channel–regulating protein, Pkd2, predominantly on the lower/dorsal side of the immotile nodal cilia (see Fig. 1B) (*29*, *31*, *32*). Activation of Pkd2, triggering *Dand5* gene degradation, which starts left-right symmetry breaking, was found to happen primarily on the left side of the node because of a higher tensile strain experienced by the dorsal side of the immotile cilia resulting from the cilia bending to the ventral side of the node (Fig. 1B). Relatively, a very low strain is experienced by the dorsal side of the right-side cilia, resulting from their less bending toward the ventral side. Asymmetric bending of the cilia was found to be caused by the nodal flow (*29*, *33*); however, the origin of the asymmetry in flow was not investigated. Note that, following the convention in this field, in the schematic of Fig. 1B and in all representations here, the node is seen from the posterior side, and thus, the left side of the node is on the viewer’s right.

Verification of numerically simulated nodal flows and the subsequent outcomes has been hampered either by the limited data because of low resolution available from the existing in vivo flow measurements or by the fact that data were obtained under conditions physiologically dissimilar to those in the embryonic node, like the flow traced in an open node with the Reichert’s membrane removed (*3*, *5*, *16*). Shields *et al.* (*34*) introduced the only artificial experimental embryonic node up to now by integrating magnetically actuated artificial cilia in a fluid cell. Contrary to the flows predicted by simulations (*15*, *21*–*23*), the flow observed in this artificial node consisted of a unidirectional leftward flow in the lower part, right above the cilia, and a returning rightward flow in the upper part of the node, along with a mixing flow in the region below the cilium tips. However, this experimental model differed from the physiological embryonic node by containing a highly dense cilium carpet, unlike the more sparse cilium density in an embryonic node, and the cilia were actuated with a small TCM of ~7° tilt, different from the extreme bending the nodal cilia typically undergo.

Because of the lack of suitable in vitro experimental models of the embryonic node and the simplifications inherent in the numerical models, there is still no consensus on the validity of the hypotheses about the role of nodal flow on the left-right asymmetry development in vertebrates. An experimental artificial embryonic node has not been accomplished because of the following challenges: mimicking the complex motion exhibited by extremely small nodal cilia, involving large deformations; the intricacy of producing such cilia in a very small space of a few hundred micrometers; and integrating them in a closed cavity in a way that allows for cilium actuation and uninterrupted visualization simultaneously. Here, using a template-based artificial cilium fabrication method, we developed an experimental artificial embryonic node that mimics the physiological node accurately by the precise control of biological parameters like node shape, cilium distributions, and large cilium deformability, combined with a detailed flow analysis by particle tracing and high-resolution particle image velocimetry (PIV). The cilia are actuated using a magnetic actuation method to mimic the large-deflection TCM of nodal cilia while allowing for an uninterrupted recording of the cilia and fluid motion. We supplement the experimental data with high-resolution computational fluid dynamics simulations that combine motile cilia with passive (primary) cilia, allowing for a detailed three-dimensional description of fluid flow, ciliary deflection, and particle transportation by convection and diffusion. Our combined system provides a detailed study of nodal flow patterns and the emergence of mechanical and chemical fluid flow–induced asymmetric response, establishing a versatile testbed to resolve the origin of the breaking of left-right symmetry in vertebrates.

## RESULTS

### Artificial embryonic node with magnetically actuated artificial cilia

Our artificial cilium fabrication process uses commercially available polycarbonate track-etched (PCTE) micro/nanoporous membranes as sacrificial molds, of which the pore size determines the cilium diameter, while the membrane thickness determines the cilium length (*35*, *36*). Given that the pores are randomly distributed over the membrane, the artificial cilia are randomly distributed as well, like in real embryonic nodes. The magnetic polymer, of which the cilia are made, is prepared by synthesizing nanomagnetic particles and coating and dispersing them in copolymers of dimethylsiloxane (see text S1). To mimic the embryonic node with motile cilia on the bottom surface of the node as shown in Fig. 1C, the PCTE mold is placed on a cured polydimethylsiloxane (PDMS) layer and filled with the uncured magnetic material containing 38 ± 2 wt % of the magnetic particles. The material is cured in a vacuum oven and then washed in chloroform to completely remove/dissolve the PCTE mold while leaving all the cilia attached to the transparent PDMS base [see Fig. 1 (C and D)]. The artificial cilia have a nominal radius of *r* = 1 μm and a length of *L* = 23 μm (see Fig. 1E), which means that they are four or five times larger than nodal cilia but have a similar aspect-ratio (*L*/*r*). The main reason for the selected cilium size is that it is the lower limit for resolving the cilium motion using a light microscope with the entire node within the field of view. The areal density of the randomly distributed artificial cilia is 1000 mm^−2^, which means that the average distance between cilia is 31 μm (see Fig. 1E).

Parallel to the cilium fabrication process, nodal cavities of different shapes—triangular, square, and circular—and different depths are fabricated using the soft lithography process (see Fig. 1D and figs. S1 and S2). Before integrating the cilia in the nodal cavities, the nodes are filled with a fluid containing fluorescent tracer particles with a diameter of 1 or 2 μm for the flow visualization and its analysis. The extraembryonic fluid in the artificial node is mimicked by an aqueous Newtonian fluid with a dynamic viscosity (η) of 2 × 10^−3^ Pa·s and a density (ρ) of 1040 kg/m^3^ (see fig. S3). The cilium layer and the nodal cavities are integrated/paired by holding the parts tightly against each other between two glass slides each 1 mm thick and sealing this stack at the ends (Fig. 1C). Details of the fabrication processes are given in text S2, table S1, and fig. S1, whereas the setup used is shown in fig. S4. The typical lateral size of our nodal cavities is 500 μm, i.e., around five times larger than real embryonic nodes. This means that the artificial node, containing cilia with a length of 23 μm and a radius of 1 μm spread sparsely like in the mouse embryonic nodes, are a four- or fivefold enlargement of real nodes. The number of motile cilia in the artificial is similar to that in real nodes, i.e., 100 to 200 (*6*). We varied the width-to-depth ratio (*w*/*d*) of the nodes corresponding to the wide range of 3 to 10 reported for embryonic nodes (*4*, *15*); specifically, three different depths (*d*) of 170, 90, and 50 μm were chosen for the three shapes corresponding to *w*/*d* ratios of 3, 5.5, and 10, respectively (see fig. S2). Figure 1D shows a top-view image of one of our circular artificial embryonic nodes.

To achieve a TCM of the magnetic artificial cilia, we designed a two-magnet arrangement as shown in Fig. 1F. Both magnets are placed off-center with respect to the artificial node, and the bottom magnet undergoes a 360° rotary motion about the vertical axis, whereas the top magnet is held stationary. The off-center configuration allows the light from an illumination source positioned below the node to pass through for uninterrupted cilium and flow visualization from the top, whereas the top magnet skews the magnetic field gradually from a parallel to perpendicular alignment and back to a parallel alignment as the bottom magnet completes one rotation (see Fig. 1F). Given that the artificial cilia tend to align continuously with the magnetic field, this gradual cyclic transition of the field direction generates a TCM of different cone tilting angles (θ) and opening angles (ψ) depending on the relative position between the two magnets and the cilia under actuation (see Fig. 1G). Using a COMSOL finite element model, we analyzed the variation in magnetic field intensity and direction resulting from the rotating magnet and selected a region for producing a typical TCM where the cilia experience large deflection so they move close to the surface during their backward stroke, aligning the cilium tip nearly parallel to the surface, therefore attaining a maximum angle of almost 90° (see fig. S5). The maximum cilium deflection angle θ + ψ with respect to the cilium base (see Fig. 1G), attained by such large deflections, takes a maximum value of around 50°, which is pragmatically optimal for generating maximal flow in the artificial embryonic node (*22*). Our artificial cilia are therefore actuated to perform a TCM with θ = ψ ≈ 25° [see Fig. 1 (G and H) and movie S1].

### Nodal flow exhibits specific patterns with multidirectional and unidirectional components

**Fig. 2.**
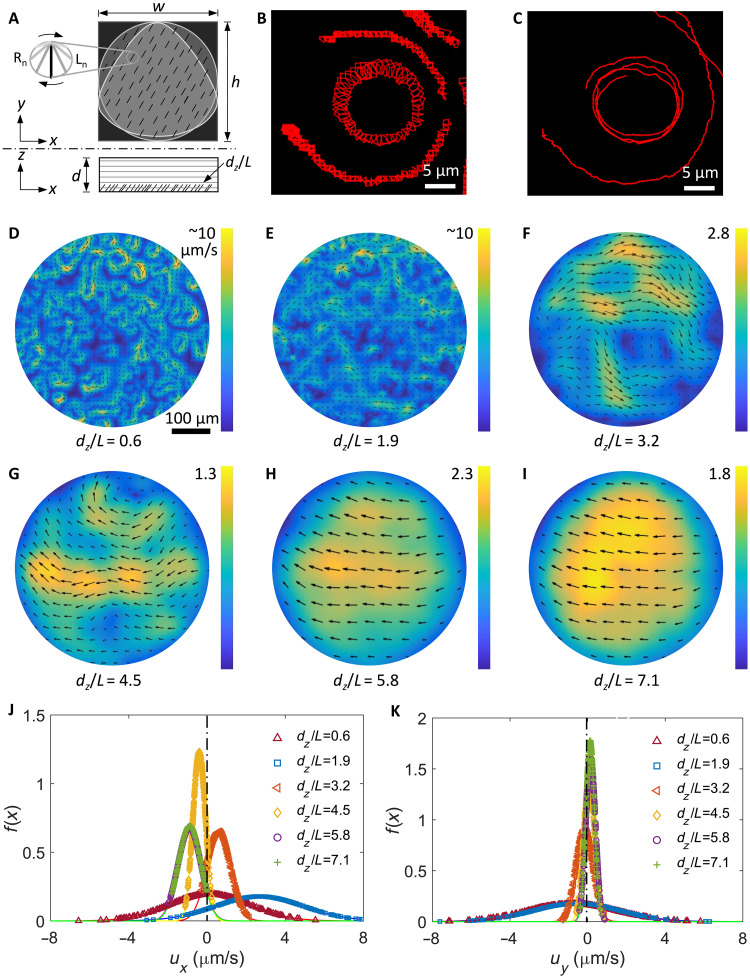
Nodal flow characterization in a circular artificial embryonic node with aspect ratio *w*/*d* = 3 (*w* = 500 μm and *d* = 175 μm), actuated at ω = 10 Hz. (**A**) Schematic representation of top and side views of the three node shapes (triangular, square, and circular) with a constant *w* and *h* = 500 μm considered in the present study. The triangular node *h* is shorter by 35 μm because of being a transformation of a circle (*22*). The schematic includes the top view of a cilium undergoing TCM, where L_n_ is the nodal left and R_n_ is the nodal right. The fluid flow characterization analysis within the node is carried out in six equidistant layers distributed over the full node depth. (**B**) Loopy motion of a particle trapped inside a cilium vortex, at *d_z_* = 15 μm, i.e., *d_z_*/*L* = 0.6, of the circular artificial embryonic node (see also movie S2). Each loop in the tracks corresponds to one cilium rotation. The particles are traced using an Olympus objective lens (MPLFLN 20xBD; focal depth, 5.2 μm), using a high-speed camera (Phantom VEO 1310L). (**C**) Loop-averaged displacement of the particles shown in (B) without the loopy part. The time-averaged motion of the fluid in all six traced layers is shown in fig. S7 and movie S4. (**D** to **I**) Color and velocity-vector maps of the fluid flow in the six observation layers, obtained from time-averaged PIV analysis. The maps are a better representation of the fluid motion than the particle traces because of the radial drift motions shown in (C) (see description in text S4). (**J** and **K**) Normal distribution function [*f*(*x*)] plots of *u_x_* and *u_y_*, fitted to the measured data, corresponding to the time-averaged velocity maps shown in (D) to (I). Movie S5 shows the nodal flow.

We first present results for a circular node with *w*/*d* = 3 and a total number of cilia *N*_c_ = 158 (Fig. 2). The circular shape is chosen for its feasibility in modeling and simulating the nodal flow; we will see later that the node shape does not influence the nodal flow characteristics. The cilia are actuated at a nodal frequency (ω) of 10 Hz with the direction of the effective/forward stroke toward the nodal left (see Fig. 2A), and the flow is traced in six equidistant layers at different depths *d_z_* = 15, 45, 75, 105, 135, and 165 μm corresponding to *d_z_*/*L* = 0.6, 1.9, 3.2, 4.5, 5.8, and 7.1, respectively (see Fig. 2A). The bottom traced layer is around midway between the cilium base and its tip with *d_z_*/*L* = 0.6. Given that the TCM of a cilium has a forward and backward stroke, the former being more effective than the latter, the fluid close to each cilium, within the cilium vortex, undergoes a spiral recirculatory motion, called “loopy drift” previously predicted through simulations (see Fig. 2B and movie S2) (*21*, *37*). The loopy motion transitions rapidly from fast recirculatory motion, closer to and around the cilium, to slow directional motion, further away from the cilium, referred to as “radial drift” (see Fig. 2B and movie S2) (*22*). Both these motion types extend to a height of around 2*L*, as observed in the second traced layer of *d_z_*/*L* = 1.9. With a number of cilia performing TCM, the loopy motion remains preserved throughout the volume of the node (see fig. S6 and movie S3). The generated net flow in the node is a combined effect of the radial drift motion resulting from all the cilia positioned randomly throughout the node. The time-averaged nodal flow is obtained by averaging the flow over each loop, revealing a streamlined flow as shown in Fig. 2C, fig. S7, and movie S4. Multiple inner circles represent the higher fluid velocity inside the cilium vortex compared to the slow-moving particle outside the vortex.

The nodal flow captured by the tracer particles, used for the PIV analysis, represents an advective flow as indicated by a high Péclet number (Pe) calculated in text S3. The steady-state PIV analysis is carried out using a Matlab toolbox (see text S4). In the bottom-most layer positioned below the cilium tip (*d_z_* = 15 μm, *d_z_*/*L* = 0.6), shown in Fig. 2D, the time-averaged PIV result shows a multidirectional flow, the specifics of which are determined by the particular spatial distribution of the nodal cilia. The multidirectional flow is further analyzed by plotting the distribution of the horizontal (*u_x_*) and vertical (*u_y_*) components of the velocities in all six layers throughout the node, and the data can be fitted well by normal distribution functions [see Fig. 2 (J and K)]. At the lowest layer (*d_z_*/*L* = 0.6), both *u_x_* and *u_y_* distribution functions are centered very close to the origin; hence, there is only very small average flow in any direction, which implies that the flow in the lowest layer may exhibit mixing but has limited or no net directionality. Flow profiles above the cilium tips obtained at *d_z_*/*L* = 1.9 and 3.2 show a smooth coarsening of the time-averaged flow patterns and a global flow that is still multidirectional but has a net leftward flow [see Fig. 2 (E and F)]. The transition is apparent in the *u_x_* distribution plots in Fig. 2J, where the peaks shift away from the origin to positive values, indicating a net mean leftward flow. Figure 2K shows that the *u_y_* distribution peaks for *d_z_*/*L* = 1.9 and 3.2 are at the origin, so there is minimal or no net *y*-directional flow. Given that the node is a closed cavity, the resultant leftward flow at *d_z_*/*L* = 1.9 and 3.2 must be compensated by a rightward return flow elsewhere, and the transition happens within a thin layer between *d_z_*/*L* = 3.2 and 4.5. At a height of *d_z_*/*L* = 4.5, an average return flow with a relatively smooth rightward direction is observed (see Fig. 2G). A more uniform directional net rightward flow develops further up at *d_z_*/*L* = 5.8 and *d_z_*/*L* = 7.1, as shown in Fig. 2 (H and I, respectively). The predominantly rightward flow in these layers is clearly visible in the *u_x_* distribution plots where almost all the data points have a negative value (Fig. 2J). The *u_y_* distribution again shows a symmetric distribution around the origin, i.e., there is no net flow in the *y*-direction, and the width of the distribution function is smaller than in lower layers, indicating less spread in values (see Fig. 2K). A high-resolution movie of the measured flow patterns in all the layers, made possible by the two-magnet arrangement, is shown in movie S5.

### Nodal flow pattern remains conserved for different node shapes but not for node depth

For the nodes with *w*/*d* = 3, we found that the overall nodal flow pattern, characterized by the time-averaged PIV maps and *u_x_* and *u_y_* distributions shown in Fig. 2, remains essentially similar for nodes with square or triangular shapes, as shown in figs. S8 and S9 and movies S6 and S7, respectively. These nodes also have different cilium distributions that affirm the independence of the nodal flow pattern on the exact cilium distribution. In addition to nodal shape and cilium-distribution independence, the flow pattern inside the node is independent of the tilting (θ) and opening angles (ψ) of the TCM exhibited by the cilia, as shown and described in fig. S10 and movie S8. A special case with zero tilting angle (θ = 0) and ψ > 0 would lead to a fundamentally different flow pattern that only produces a rotational nodal flow in the *xy* plane and no leftward or rightward flow in the lower and upper part, respectively, of the node (*15*). This case is therefore excluded from our analysis of the artificial embryonic node. Furthermore, identical flow patterns invariable of the node shape, cilium distribution, or TCM angles stay consistent over a large actuation frequency (ω) range of 1 to 50 Hz, which unveils the highly conserved nature of the typical flow inside the embryonic node (see fig. S11 and movie S9). The conserved flow pattern is a consequence of the low Reynolds number (*Re*) flow generated in the node. For a typical ω = 10 Hz, the highest observed velocities of around 35 μm/s near the cilium tip and around 250 μm/s in a simulated flow (as we will see later) and the representative value of around 3 μm/s away from the cilia correspond to very low *Re* values of 3.2 × 10^−3^, 2.3 × 10^−2^, and 2.7 × 10^−4^, respectively (see text S5). At ω = 50 Hz, *Re* still takes a low value of around 10^−2^. At such low *Re*, the flow is dominated by viscous effects and inertial effects are absent; hence, the mean fluid velocity *u_x_* at different nodal depths increases linearly with increasing ω, as shown by measured data in fig. S12.

**Fig. 3.**
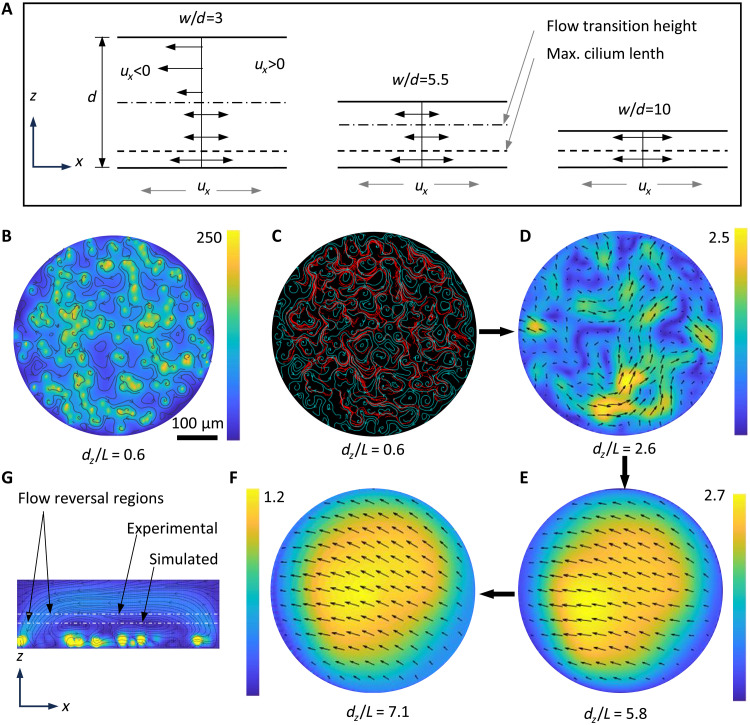
Nodal flow schematics and simulated flow. (**A**) Schematic representations of the measured cross-sectional mean flow profiles in the nodes with different aspect ratios *w*/*d*, with double-headed arrows indicating a multidirectional net flow and single-headed arrows indicating a directional net flow. The placement of the double-headed arrows with respect to the central vertical line in the schemes indicates a net leftward (more positive) flow, a net rightward (more negative) flow, or no net (symmetric) flow. (**B**) Simulated time-averaged flow of the bottom-most layer at *d_z_*/*L* = 0.6 with the cilium distribution and nodal dimensions exactly the same as those of the experimental node shown in Fig. 2 (D to K) with *w*/*d* = 3. The streamlines indicate the multidirectional nature of the flow. (**C**) The simulated streamlines show a close match with the experimental particle tracks, superimposed here, validating the simulated nodal flow pattern. (**D** to **F**) The transition of the nodal mean flow from multidirectional flow in the bottom part to the directional and rightward flow in the upper part [shown in Fig. 2 (D to I)] is closely replicated in the simulations, as shown by these simulated color and velocity-vector maps of the time-averaged flow. A side-by-side comparison of the simulated flow with the observed flow in the node is shown in fig. S16B. (**G**) Simulated cross-sectional flow averaged over one complete rotation of the cilium cycle. The height within the node at which the flow reversal happens is indicated by the horizonal line for both the simulations and experiments.

For shallower nodes where the ratio *w*/*d* changes from 3 to 5.5 to 10, the flow patterns show a marked change. For a node with *w*/*d* = 5.5, the flow patterns in the lowest layers are similar to those in the deeper node with *w*/*d* = 3 [see fig. S13 (A and B)]. However, the net rightward return flow for the shallower node is limited to a small region near the top of the node, and the flow pattern there is much less uniform and shows quite some multidirectionality compared to the deeper node (see fig. S13C and movie S10). These observations are confirmed by the normal distribution plots of *u_x_* and *u_y_* at different depths of the node [see fig. S13 (D and E)]. With a further decrease in nodal depth to *w*/*d* = 10, the top return flow completely vanishes and a multidirectional flow develops throughout the full nodal depth [see fig. S13 (F to H) and movie S11]. Although the flow pattern changes with *w*/*d*, it stays conserved over different node shapes for a given *w*/*d* ratio, as shown in figs. S14 and S15 and movies S12 to S15 of rectangular and triangular nodes with *w*/*d* = 5.5 and 10. Taking the measured mean *u_x_* at different *d_z_*/*L* heights for all the three nodes with aspect ratios *w*/*d* = 3, 5.5 and 10, the cross-sectional net nodal flow can now be schematically represented as shown in Fig. 3A. Only the deepest node with *w*/*d* = 3 shows a directional net flow above the transition line, whereas the shallowest node with *w*/*d* = 10 does not show any net flow at all. Hence, only the node with *w*/*d* = 3 shows a flow pattern that matches with the observed flow in the mouse node (*5*). Nodes with higher *w*/*d* ratios of 5, previously considered for simulating the nodal flow (*15*), and 10, in a limiting case (*4*), therefore do not faithfully represent the nodal geometry. On the basis of the in vivo geometrical and operational parameters used in our study, the artificial embryonic node flow pattern transitions from out-of-plane to in-plane motion at *w*/*d* > 10 or *L*/*d* > 0.5. It would be interesting for future studies to explore the interdependence of the various parameters in this confined fluid-flow system, potentially described by an appropriate dimensionless number.

### Nodal flow is predicted by a fluid-structure interaction computational model

For a detailed analysis of the node with *w*/*d* = 3, the flow pattern observed is further compared and analyzed using a computational model using a fluid-structure interaction framework (see text S6) (*38*, *39*). The magnetic artificial cilia are modeled as rigid bodies, supplied with kinematically described TCM motion governed by a time-varying magnetic field extracted from a COMSOL Multiphysics model of the two-magnet arrangement used in actuating the cilium structures (see fig. S16A and movie S16). In the bottom layer at *d_z_*/*L* = 0.6, the simulated time-averaged flow reveals a multidirectional flow similar to the measured flow pattern with peak velocities at the tip of each cilium (see Fig. 3B). The highest simulated velocity of 250 μm s^−1^ exists at the cilium tip within the cilium vortices rotating at 10 Hz, captured because of the fine size of the simulation mesh. A lower velocity of 33 μm s^−1^ obtained through the PIV analysis (see Fig. 2D) is due to the limited number of PIV particles possible to get trapped within the cilium vortices, which cannot capture the large velocity gradient radiating away from the cilium tip. A comprehensive validation of the computational model is performed by superimposing the flow streamlines extracted from the simulated flow with the particle trajectories as shown in Fig. 3C. In addition, the multidirectional nature of the flow in the bottom part and the directional net flow in the upper part of the artificial embryonic node is well predicted by the simulations, as shown in Fig. 3 (C to F) and fig. S16B. Following validation, the flow reversal within the node is investigated by analyzing the simulated cross-sectional mean flow patterns (see Fig. 3G and movie S17), which are almost impossible to measure experimentally.

The simulated cross-sectional flow averaged over one complete rotation of the cilium cycle shows the net flow transition and its reversal in the node. A small difference in the flow reversal height obtained in the simulations to the experimentally observed height is most likely due to the rigid cilium body assumption applied in the simulations compared to the flexible bending of the cilia. The fluid moves upward (or ventrally) along the nodal left region at a steeper angle than it moves down (or dorsally) on the nodal right side to complete the net flow circulation within the node. The flow field information obtained through the simulations is further used for studying the mechanism of symmetry breaking in an embryo.

### Asymmetric flow between left and right node sides causes distinct primary cilium bending

**Fig. 4.**
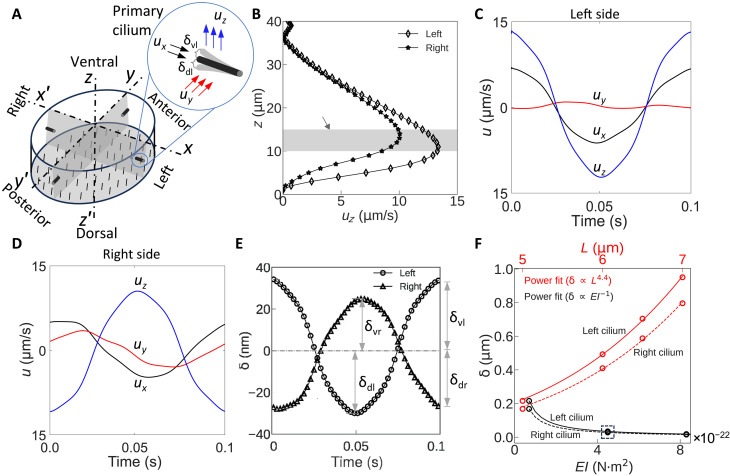
Immotile cilium deflection. (**A**) Schematic representation of the immotile cilium location on the left, right, front/posterior, and back/anterior of the embryonic node. The zoomed-in section represents the three velocity components of the time-dependent fluid flow experienced by the immotile cilia and the deflection caused by the *z*-component of the flow field. In this figure, all dimensions and velocities are scaled down linearly 4.5 times compared to the previous figures so that the results can be interpreted directly in terms of real embryonic nodes, which are four or five times smaller than our artificial node. (**B**) Magnitude of the maximum *z*-component velocity (at the tip of the immotile cilia) along the depth (*d*) of the scaled-down node. The shaded region, showing peak velocities, is considered for the immotile cilium location within the node for their maximum responsiveness. (**C** and **D**) Variation of the scaled-down time-resolved peak velocity components in all three directions experienced by the left and right immotile cilia, not necessarily at the cilium tips. Peak fluid velocities experienced by the anterior and posterior immotile cilia are shown in fig. S19 (A and B). (**E**) Tip deflection of the immotile cilia on the left and right sides of the node with an average cilium length of 5 μm, a diameter of 200 nm, and a flexural rigidity of 4.5 × 10^−22^ N·m^2^. The anterior and posterior immotile cilia show a similar deflection trend (see fig. S19C). (**F**) Maximum tip deflection δ as a function of immotile cilium length *L* (red) keeping the cilium flexural rigidity *EI* at a minimum measured value (*29*) of 0.7 × 10^−22^ N·m^2^ and as a function of *EI* (black) at a constant cilium length and diameter of 5 μm and 200 nm, respectively. Tip deflection enclosed in the square corresponds to conditions in (E).

To investigate the two-cilium hypothesis (mechanosensing), we evaluate the extent of deflection of immotile/primary cilia present along the outer region of the node under the influence of the nodal flow. To this end, the flow variation along the left and right vertical sides of the node (the crown), in the region where the primary immotile cilia are located, as shown in Fig. 4A, is extracted from the simulations (see Fig. 4B). As the distribution/location of the immotile cilia around the sides of the physiological node has not yet been quantified, we consider them to populate the region where they experience the highest flow velocities in order for them to undergo maximum deflection (see Fig. 4B). The time-dependent flow experienced by the immotile primary cilia on the left and right sides of the node varies in magnitude and direction over each cycle of cilium rotation because of the forward and backward stroke of the TCM, as shown in movie S18 and by the computational results in Fig. 4 (C and D). The most substantial velocity component is in the *z*-direction, i.e., along the dorsal-ventral axis. Therefore, the immotile cilia will bend primarily in the *z*-direction, as observed in the mouse node (*29*). The cyclic variation of *u_z_* generating the oscillatory motion of the immotile cilium is shown in fig. S18. The magnitude of the simulated nodal vertical flow *u_z_* is larger on the left side compared to the right side of the node [see Fig. 4 (C and D)]. The stronger flow on the left side is due to the steeper upward movement of fluid on its left side (see Fig. 3G) caused by the stronger flow generated during the effective stroke than during the recovery stroke of the TCM of the nodal cilia. Using a COMSOL model (see text S7 and fig. S17A), we analyze in detail the deflection of the immotile cilia resulting from the local flow around the cilia, taking full account of solid-fluid interaction. To do so, the time-dependent, cyclic flow velocities at the immotile cilia extracted from the full nodal system are applied to a representative immotile cilium of average dimensions. This results in the oscillatory motion of the cilia with the amplitude on the left side being larger than on the right side in both the dorsal and ventral directions, with a larger deflection toward the upper/ventral side (δ_vl_ − δ_dl_ > 0), as observed in the mouse node (see Fig. 4E and movie S19) (*29*). The magnitude of the cilium deflection is primarily decided by the cilium length *L* and its flexural rigidity *EI* (see Fig. 4F) that shows that the maximum tip deflection δ increases with *L* and decreases *EI*. The tip deflection increase with the length follows a power law δ = *L*^4.4^, as explained in text S8. Only at a maximum reported length of 7 μm and at a minimum reported flexure rigidity of 0.7 × 10^−22^ N·m^2^, the asymmetric tip deflection is comparable to the deflection observed in the mouse node where the cilia on the left side undergo an average deflection of 1.2 μm toward the ventral side (*29*). Figure 4F shows that the immotile cilia on the left (solid line) and right (dotted line) sides show asymmetric tip deflection values of around 1 and 0.8 μm, respectively, under these conditions.

Together, we can now evaluate the possible mechanism behind the activation/deactivation of the mechanosensitive cation channel–regulating protein Pkd2 on immotile cilia to break the symmetry during the embryonic development of vertebrates. First, the deflection of immotile cilia in the mouse node shows an oscillatory motion (*29*), as also confirmed in our artificial embryonic node, and it arises from the loopy motion of the nodal flow (Fig. 4D). A highly regular spiral/loopy nodal flow is due to the strictly in-phase rotation of all the cilia in the artificial embryonic node compared to the naturally random phase difference in the mouse node, which may produce a less regular loopy nodal flow. The immotile cilia on the left and right sides of the artificial embryonic node bend more toward the ventral side and dorsal side, respectively, which is key to the asymmetric activation of the primary cilia in the physiological node (*29*). With the presence of Pkd2 on the lower/dorsal side of the cilia as reported in (*29*), the larger upward/ventral deflection of the immotile cilia on the left side, compared to the cilia of the right side, exerts a higher tensile strain on Pkd2 present on the left cilia, therefore imparting precedence to their activation followed by the consequent signaling cascade to develop the asymmetry. In conclusion, our results support the validity of the two-cilium hypothesis.

On the basis of the asymmetric nodal flow development in a geometrically symmetric node, we can extend our observations to the embryonic node of zebrafish, called Kupfer’s vesicle, which has a circular cross-sectional shape with a rotational nodal flow (*31*). The motile cilia in the node generate a net counterclockwise flow, making the immotile cilia on the left side of Kupfer’s vesicle undergo higher deflection compared to those on the right side (*31*). Hence, a symmetric geometry producing an asymmetric flow is true for both fish and mouse embryos. In future work, our analysis can be extended to Kupfer’s vesicle to study this in detail. In addition, the representation of the node shown schematically in Fig. 1B and used in our work has sidewalls vertical to the node base unlike the real node, which has curved sidewalls as apparent in Fig. 1A. The curved shape does not change the flow pattern in a notable manner, as indicated by an earlier study (*40*), and our results confirm that flow asymmetry does emerge in a node that is symmetric with respect to the plane through its *yz* plane. Hence, while the magnitude of the flow and the immotile cilium bending may differ between the curved and vertical sides of the node, the asymmetry will still persist, which is the essential implication of the nodal flow pattern observed in our artificial embryonic node.

### A net particle gradient is generated because of convection and diffusion effects

**Fig. 5.**
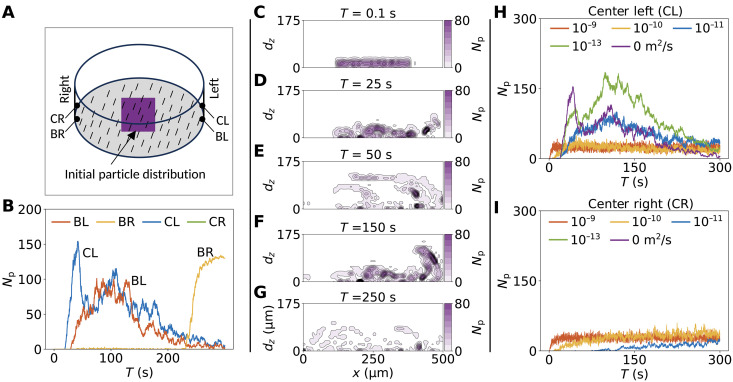
Convection and diffusion of particles by nodal flow. (**A**) Schematic illustrating the initial position of particles spread over an area of 250 by 250 μm and a height of 25 μm for a circular node with width *w* = 500 μm and depth *d* = 175 μm (i.e., *w*/*d* = 3). The regions of interest, or “receptor locations,” where particles are tracked on the left and right sides of the node include the following: bottom left (BL), bottom right (BR), center left (CL), and center right (CR); the latter two are located at half the node depth. (**B**) Time evolution of particle distribution at the various receptor locations for purely convective particle motion. (**C** to **G**) Contour plots in the plane through all receptor locations, showing particle distribution at different time points, 0.1, 25, 50, 150, and 250 s, for purely convective particle motion (see movie S20). The color map represents the number of particles *N*_*p*_ in a 20-μm-thick slab. (**H** and **I**) Time evolution of the particle distribution at CL and CR for various particle diffusion coefficients (m^2^ s^−1^) and for purely convective particle motion where *D* = 0.

To investigate both chemosensing hypotheses (because of a morphogen gradient and because of NVP transport), we numerically model the convection and diffusion of massless particles that have biologically relevant diffusion coefficients. Details of the numerical approach are given in text S9. Following the literature (*5*, *18*, *26*), we position the particles initially at the center of the base of the artificial node within a stack of 250 μm by 250 μm by 25 μm, as shown in Fig. 5A. Previous studies (*5*, *15*, *18*, *40*) suggest that morphogens are detected primarily on the bottom surface of the node, while NVPs have been suggested to rupture (releasing morphogens) near the perinodal (immotile) cilia or nodal crown cells at the bottom near the node walls (*18*, *40*). On the basis of these, we track the accumulation of particles at four key sensing sites shown in Fig. 5A. The time evolution of the particle distribution under purely convective particle motion at different receptor sites is presented in Fig. 5 (B to G) and movie S20. Initially, all particles are concentrated at the center of the node (Fig. 5C). As the flow begins, particles start migrating toward the nodal left. By 25 s, the particles begin to accumulate near the central and bottom left receptor sites (Fig. 5, B and D). A notable increase in particle density near the central left site is observed around 50 s (Fig. 5, B and E), while the bottom left receptor reaches a maximum particle density at 100 s (Fig. 5B). Given that our simulations do not incorporate particle degradation or deactivation upon reaching receptor sites, particles continue to circulate within the embryonic node. Consequently, over time, the particle density at the left receptor sites decreases. As circulation continues, the particles arrive at the right side of the node, resulting in an increasing particle concentration at the bottom right receptor location (Fig. 5, B and G). Other receptor sites, such as the central right, show almost no particle deposition. The observed spatial and temporal asymmetry in particle concentration between the left and right receptor locations suggests a potential mechanism for symmetry breaking within the node.

Morphogens, NVPs, and other signaling proteins exhibit diffusion coefficients ranging from 1 × 10^−13^ to 1 × 10^−10^ m^2^ s^−1^ (*5*, *15*, *26*, *40*). To assess the relative influence of convection and diffusion on particle transport, we extend the analysis by systematically varying the diffusion coefficient in the range of 1 × 10^−16^ to 1 × 10^−9^ m^2^ s^−1^. Figure 5 (H and I) shows the time evolution of particle distribution near the central left and right receptor sites under varying diffusion coefficients. Figure 5H shows that the peak particle accumulation near the central left site decreases as diffusion increases, shifting from localized deposition to global spreading. Similarly, Fig. 5I reveals that higher diffusion coefficients result in a uniform distribution over time, while minimal accumulation occurs at the central right site for lower diffusion values. These results highlight a critical diffusion threshold between 1 × 10^−10^ and 1 × 10^−11^ m^2^ s^−1^, consistent with biological observations (*5*, *18*). Proteins that fall within this critical range and considered previously in an in vivo study of left-right asymmetry development (*5*) are soybean trypsin inhibitor, bovine chymotrypsinogen A, and carbonic anhydrase, with molecular weights of 21.5, 23, and 31 kDa and diffusion coefficients of 1 × 10^−10^, 9.5 × 10^−11^, and 11 × 10^−11^ m^2^ s^−1^, respectively. When the diffusion coefficient is 1 × 10^−10^ m^2^ s^−1^ or higher, diffusion dominates transport dynamics, leading to a homogenized distribution. In contrast, when the diffusion coefficient is 1 × 10^−11^ m^2^ s^−1^ or lower, the distribution more closely resembles the case dominated by convection (Fig. 5B). Additional analyses of diffusion effects on particle distributions are provided in fig. S20.

These findings demonstrate that, for diffusion coefficients lower than 1 × 10^−11^ m^2^ s^−1^, convection dominates and the particles accumulate predominantly at the bottom left receptor site within the first 200 s. This asymmetric particle distribution points to chemosensing as a potential driver of left-right symmetry breaking in vertebrates, in agreement with prior experimental (*3*, *5*, *18*, *26*) and computational (*15*, *40*, *41*) studies. The time period of 2 to 3 min, during which particles predominantly accumulate at the bottom left receptor, also aligns with the findings in (*5*). In addition, our results reveal a fast and strong accumulation of particles at the central left site, where the left immotile cilia are located.

## DISCUSSION

In summary, the comprehensive nodal flow analysis made possible by our artificial embryonic node provides the solid conclusion that the nodal flow is independent of nodal shape, and an asymmetric flow pattern is generated in a node that is geometrically symmetric to the plane through the anterior-posterior and dorsal-ventral axes. While the shape is not crucial, we found the width-to-depth ratio *w*/*d* to be the most sensitive parameter of the node. For *w*/*d* = 3, but not for higher aspect ratios, we found a unidirectional rightward net return flow in the top region of the node, which has also been observed in in vivo studies; however, in the few studies that report the aspect ratio, values are close to *w*/*d* = 5.5 (*4*, *15*), for which our artificial node shows a multidirectional net return flow and not a uniform one. Given that the in vivo measurements in the embryonic node involve certain modifications of the node, like removing Reichert’s membrane, more measurements will be needed in the future to establish the range of *w*/*d* ratios of embryonic nodes with a higher degree of certainty. Regarding the true nature of the fluid flow within the node, it is evident that only the loopy or pulsatile motion we measured and simulated will induce oscillatory deformation of the immotile cilia. Clearly, high frame-rate flow tracing, preferably in the upper part of the node, is key to identifying the extent to which the fluid motion is loopy or pulsatile in real nodes. Toward testing the mechanosensing hypothesis, the maximal tip deflection of cilia depends strongly on cilium length (to the power of 4.4). For the stiffnesses reported for a mouse embryonic node (*29*), our results show that a length of 7 μm is needed to reach reported deflections of 1.2 μm (*29*) for triggering the cilium sensory activation. The only study so far of the immotile cilium length distribution (in the mouse node) suggests that most of the cilia have a length of around 5 μm but that 5% have a length of around 7 μm (*5*). Given that a single immotile cilium, undergoing appropriate oscillatory motion, can be sufficient to trigger the asymmetric response (*29*, *31*), our study suggests that only a few cilia or even a single cilium of the longest length with the lowest flexural rigidity participates in the symmetry breaking process. Future work is needed to study in more detail the immotile cilium length, width, and stiffness distribution to substantiate the mechanosensing response of the node. The measurements should be done in vivo as the dried samples used for scanning electron microscopy are found to alter the original cilium dimensions (*5*).

Furthermore, our analysis of the spreading of particles within the node, which are released from the center of the base of the node, shows spatial and temporal asymmetry. Within the first few minutes, the particles accumulate on the nodal left, provided that their diffusion coefficient is not larger than 1 × 10^−11^ m^2^ s^−1^, which is valid for morphogens, NVPs, and other signaling proteins. Our experimental artificial embryonic node combined with numerical simulation therefore offers detailed insight into the complex nodal flow process and its evolution toward the asymmetry development during embryonic development; our results support both the mechanosensing and chemosensing hypotheses from a fluid mechanics point of view. Combining our observation of the asymmetry in deflection of the immotile cilia with our particle distribution results, we hypothesize that nature may have integrated both mechanosensing and chemosensing at the left immotile cilia to reinforce symmetry breaking. This suggests a potential synergy between the two sensing mechanisms to enhance the biological robustness of left-right asymmetry, as also speculated in (*29*).

## MATERIALS AND METHODS

The magnetic material preparation process is described in text S1, and the artificial embryonic node fabrication process is explained in detail in text S2 and fig. S1. The PIV analysis is described in text S4. Text S6 contains detailed information on the computational modeling of the nodal flow, and text S7 and fig. S17 describe the COMSOL model used to simulate the deflection of primary cilia caused by the simulated flow field. Details about the calculation of convection and diffusion of particles resulting from the simulated flow are given in text S9.

## Supplementary Materials

### The PDF file includes:

Supplementary Text S1 to S9 Figs. S1 to S20 Table S1 Legends for movies S1 to S20 References
