# Supplementary material for: Artificial embryonic node elucidates the role of flow in left-right symmetry breaking in vertebrates
Source: Sci Adv. 2026 Mar 25;12(13):eaec2328. doi: 10.1126/sciadv.aec2328 (PMC13267319; doi:10.1126/sciadv.aec2328)
Supplement: Supplementary file 1 — Supplementary Text S1 to S9 Figs. S1 to S20 Table S1 Legends for movies S1 to S20 References [file sciadv.aec2328_sm.pdf]

Science Advances
AAAS

Supplementary Materials for

Artificial embryonic node elucidates the role of flow in left-right symmetry breaking in vertebrates

Tanveer ul Islam *et al.*

Corresponding author: Patrick R. Onck, p.r.onck@rug.nl; Jaap M. J. den Toonder, j.m.j.d.toonder@tue.nl

Sci. Adv. **12**, eaec2328 (2026)
DOI: 10.1126/sciadv.aec2328

The PDF file includes:

Supplementary Text S1 to S9
Figs. S1 to S20
Table S1
Legends for movies S1 to S20
References

Other Supplementary Material for this manuscript includes the following:

Movies S1 to S20

Text S1: Magnetic material preparation process

Beginning with the synthesis of nano-magnetic particles (magnetite) to be coated with a polymer (35) followed by their dispersion in another polymer, a highly homogeneous magnetic-elastomer is prepared. The particles are synthesized by first mixing two iron salts, ferric chloride $(FeCl_3)$ and ferrous chloride tetrahydrate $(FeCl_2.4H_2O)$ in 2:1 molar concentration, and titrated with a 28% ammonium hydroxide solution in an inert environment (35,44). A polymer containing $6 - 7\,\mathrm{mol}\%$ of aminopropylmethylsiloxane with dimethylsiloxane (AMS-162 from Gelest) is added and stirred overnight to allow particle-polymer complexion. The resulting solution is washed to remove the byproducts by first settling the coated/ uncoated particles on a permanent magnet and decanting the clear solution left above. The complete removal of byproducts and the extra polymer is achieved by washing the particle-polymer complexion three times with methanol, three times with water, and then again three times with methanol. The washed content is dispersed in a chloroform solvent, where the coated particles form a stable solution with the solvent while a small quantity of uncoated particles is collected by a permanent magnet and separated from the solution. The particle-polymer complexion contains $44 \pm 2\ \mathrm{wt}\%$ of nano-magnetic particles which is fine-tuned, while being dispersed in the chloroform, to the required concentration of $38 \pm 2\ wt\%$ by adding a polymer containing $9 - 11\ \mathrm{mol}\%$ of aminopropylmethylsiloxane with dimethylsiloxane (AMS-162 from Gelest). The polymer added for concentration tuning has a low molecular weight (M) and viscosity (η) of $2000 - 3000\,\mathrm{g/mol}$ and $38 - 58\,\mathrm{cP}$ respectively compared to $M = 4000 - 5000\,\mathrm{g/mol}$ and $\mu = 77 - 116\,\mathrm{cP}$ for the coated polymer (AMS-162). The addition of the lower M tuning polymer is intended to develop a magnetic elastomer (after curing) with the lowest Young's modulus (E) for maximum cilia bending and actuation to mimic the large deformation of the nodal cilia. Using a custom-made setup, the material E is measured through a compression test to be equal to $1.0 \pm 0.2\,\mathrm{MPa}$.

Text S2: Fabrication process steps

The fabrication is completed in three major parts: cilia fabrication, SU-8 template fabrication using photo-lithography followed by nodal cavity fabrication using soft-lithography, and device integration, see Fig. S1.

Cilia fabrication steps:

- Preparing a substrate with a spin-coated PDMS layer of $200\,\mu\mathrm{m}$ thick at $300\,\mathrm{rpm}$ for $60\,\mathrm{s}$ on a glass slide of $1\,\mathrm{mm}$ thickness, Fig. S1(i). PDMS, which contains a curing agent in a ratio of $10:1$, is heat-cured at $65\ ^0C$ for $2-3\,\mathrm{hrs}$.

- Placing the PCTE mold on the cured PDMS layer to be filled with the magnetic fluid, Fig. S1(ii).

- Applying a thick layer of $\approx 100\,\mu\mathrm{m}$ of the prepared magnetic polymer and heat-curing at $120\ ^0\mathrm{C}$ for $90\ \mathrm{min}$ in a vacuum oven to avoid oxidation of the magnetite particles, Fig. S1(iii).

- Removal of the cured device from the glass substrate and washing in chloroform to detach the top magnetic layer by stress due to swelling in chloroform, dissolving the PCTE mold and releasing the cilia. The released cilia are preserved in a fluid environment (ethanol) before placing on a glass substrate during device integration, Fig. S1(iv-v).

The SU-8 template and nodal cavity fabrication involves the following steps:

- Spin coating of a negative photoresist $(\mathrm{SU}-82050)$ on a 4-inch silicon wafer using the parameters shown in Table S1 for different depths (d) of the nodal cavities, Fig. S1(vi).

- Heating the coated wafer (baking) on a hot plate by gradually increasing the temperature at $5\ ^0\mathrm{C}/\mathrm{min}$ to $65\ ^0\mathrm{C}$ and holding for a specific time given in Table S1. The temperature

is again gradually increased and maintained at $95\ ^0$C before letting the plate cool to near room temperature.

- ❏ Exposing the baked wafer to a UV dose, see Table S1, through a photomask containing features of different shapes, circular, square and triangular, and size equivalent to the artificial embryonic node width, Fig. S1(vii-viii).
- ❏ Gradual heating of the UV-exposed wafer (post-bake) at $65\ ^0$C first and then at $95\ ^0$C followed by slowly cooling back to room temperature; see Table S1 and Fig. S1(ix).
- ❏ Washing of the nonexposed photoresist by mr-DEV 600 solvent on a stirring plate at $180\ \mathrm{rpm}$ for $10\ \mathrm{min}$ and repeating the process in fresh solvent followed by washing in isopropyl-alcohol, Fig. S1(x).
- ❏ Pouring PDMS on the SU-8 mold and curing at $65\ ^0$C for $2-3\ \mathrm{hrs}$ and demolding, Fig. S1(xi-xv). Depth of fabricated nodes is measured using a profilometer as shown in Fig. S2.

Device integration steps:

- ❏ Replacing fluid around the cilia with a prepared fluid containing water, soap molecules and glycerol (99.5% from Merck) in the ratio $10:2:2$ and mixed with florescent tracer particles. The fluid exhibits a small contact angle with PDMS, due to the presence of soap molecules, required for better cilia wettability, whereas a stable dispersion of fluorescent tracer particles is achieved by tuning the density of the fluid by adding glycerol to match the density of the tracer particles, resulting in a fluid density of $\rho = 1040\mathrm{kg/m^3}$. Particles of 1 or $2\,\mathrm{\mu m}$ (FH-1056-2 and FH-1056-2 from Spherotech) are mixed at a concentration of $40\,\mathrm{\mu g}$ of particle solution to $1\ \mathrm{ml}$ of the prepared fluid representing the extraembryonic fluid. Particles with a diameter of $2\,\mathrm{\mu m}$ are used in the nodes with $w/d = 3$ while the

particles of $1\,\mu\mathrm{m}$ are used in all the other nodes. Fluid viscosity measurements are shown in Fig. S3.

- ❏ Filling of the nodal cavities with artificial extraembryonic fluid after attaching the layer to a glass slide.
- ❏ Gentle placement of the filled nodal cavities in the fluid-covered cilia under a vertical $5\,\mathrm{mm}$ diameter post with rounded end connected to a screw gauge.
- ❏ Pressing the two parts by applying single-point force through the rounded end of the post using the screw gauge. The application of a single point force avoids slipping of the two layers and therefore the detachment of the cilia from the base, Fig. S1(xvi).
- ❏ Sealing of the ends by applying a fast cure epoxy to ensure leakproof pairing of the two parts, as the soap containing fluid acts as a lubricant and bars the two polymer surfaces to adhere to each other, Fig. S1(xvii). Completely closed cavities faithfully represent a closed embryonic node.

Text S3: Péclet number

In the node, the particle transport/ movement is analyzed to verify that the tracer particle motion used for mapping the nodal flow is an advective phenomenon rather than being diffusive (i.e. Brownian motion), so that the particles follow the fluid streamlines. The Péclet number (Pe) which represents the ratio of advective to diffusive transfer over a characteristic length scale (w) (in our case, the width of the node) is evaluated for the node and is given by:

$$\mathrm{Pe} = \frac{uw}{D_0}, \tag{1}$$

where u is the characteristic velocity and D_0 is the mass diffusivity given by the Stokes-Einstein equation, $D_0 = (\mathrm{k}T)/(3\pi\eta d_p)$, in which $\mathrm{k}T$ is the thermal energy and d_p the particle diameter. For the tracer particles of diameter 1 and $2\,\mu\mathrm{m}$, D_0 is equal to $2 \times 10^{-13}\ \mathrm{m}^2/\mathrm{s}$ and $1 \times 10^{-13}\ \mathrm{m}^2/\mathrm{s}$, respectively. At a characteristic flow velocity of $1\ \mu\mathrm{m/s}$ and $100\ \mu\mathrm{m/s}$, the Pe for $1\ \mu\mathrm{m}$ is around 2290 and 229000 respectively whereas for $2\,\mu\mathrm{m}$ it is 4580 and 458000 respectively. These very large Pe values of the tracer particles indicate that the artificial nodal flow measured and the PIV analysis done strictly represents the fluid movement in the node, while Brownian motion is negligible.

Text S4: Particle image velocimetry (PIV) analysis

PIVlab, a Matlab-toolbox, is used to analyze steady-state flow in the artificial embryonic nodal. The toolbox identifies the particles in each frame as white spots on a black background. Monochrome recordings of nodal flow are processed using the 'ImageJ' software to convert the dark spots, particles, and cilia, into white spots by first enhancing the brightness and then inverting the colors. The same processing is needed for the particle tracking shown in Fig. S6 and Fig. S7 which forms the basis for choosing the suitable parameters to carry out the PIV analysis. The steady-state fluid motion, without the loopy part as shown in Fig. S7, shows a

disordered flow pattern in the lower part, while the flow tracks are linearly ordered in the upper part of the node.

In PIVlab, the "direct Fourier transform (FFT)" correlation with two passes is used for the upper layers, whereas for the lower layers four passes are used for higher resolution to capture high velocity gradients around small cilia vortices. Large interrogation areas of 64 (pass-1) and 32 (pass-2) pixels are used in the upper layers, whereas in lower layers area sizes of 64, 32, 16 and 8 pixels are used. For comparison, the artificial embryonic nodes of $500\,\mu\mathrm{m}$ width correspond to 575 pixels in each frame. Before running the PIV-analysis on the selected interrogation areas, the cilia that appear as static white spots in the bottom layers, due to their frequency matching the selected frame rate, are removed by applying the 'background subtraction' under the image preprocessing option.

To remove noise and erroneous vectors, after establishing the cross correlation, the velocity-based vector validation is used to refine the velocities. These vectors are clearly differentiable on a scattered plot of all the computed velocities. To minimize interpolation of missing velocity vectors caused by the absence of particles in certain regions, a high number of frames recorded over a time period of 30 s are analyzed for each layer. Taking the mean of all the frames yields a very accurate velocity map of the fluid flow. To address minor inconsistencies in velocity vector orientations, a smoothing function is applied as the final step in the PIV analysis process.

Since there is a significant velocity gradient around each cilium in the bottom part of the node, the smoothing function greatly suppresses these gradients while providing a more accurate map of the fluid flow away from the cilia vortices, due to the radial drift motion. To validate the results, the maximum velocities obtained through PIV analysis are compared with values derived from particle tracking analysis.

Text S5: Reynolds number

Flow generated in the node by the rotating cilia has the highest velocities at the cilia tips as being furthest from the rotating point. The velocity drops rapidly away from the cilia and the circulatory motion around the cilia captured by the tracer particles is the highest velocity the fluid experiences, see Movie S4. The velocities in these regions are considered for calculating the Reynolds number Re given by:

$$\mathrm{Re} = \frac{\rho u d}{\eta}, \tag{2}$$

where η is the dynamic viscosity of the artificial extraembryonic fluid, u is the characteristic velocity in the node, and the node height d, being the smallest geometric feature of the node, is taken as the characteristic size of the node (instead of the width w considered previously (15)). With $u = u_{\mathrm{max}} = 35\,\mathrm{\mu m\,s^{-1}}$ at $\omega = 10\,\mathrm{Hz}$ in a node of $w/d = 3$ and $d = 175\,\mathrm{\mu m}$, the maximum Reynolds number $\mathrm{Re}_{\mathrm{max}}$ takes a value of 3×10^{-3}. Since the nodal flow is due to the fluid flowing away from the circulating regions around the cilia, the Reynolds number calculated based on the velocity away from the cilia tips is a better representative for the flow in the node. With $u \approx 3\,\mathrm{\mu m\,s^{-1}}$, see Fig. 2, the nodal Re takes a very low value equal to 3×10^{-4}. Taking $\omega = 50$ Hz and $u_{\mathrm{max}} \approx 100\,\mathrm{\mu m\,s^{-1}}$ brings the maximal Reynolds number at higher beating frequency $\mathrm{Re}_{\mathrm{max}}$ to around 10^{-2}, which is still negligible compared to 1. The low Reynolds numbers justify the conserved nature and the steady state flow in the node for all used ω.

Text S6: Nodal flow modeling

The flow behavior within the artificial embryonic node is investigated using a computational fluid dynamics framework (38,39). The circular artificial embryonic node with $w/d = 3$ and cilia size and distribution that matches the node shown in Fig. 2 is considered here. The cilia, with a diameter of 2 µm and a height of 23 µm, are modeled as rigid rods. The cilia exhibit kinematically described motion, which is governed by a time-varying magnetic field under the assumption that the cilia fully align with the magnetic field direction at each instant, see Fig. S16(A). The magnetic field is obtained by simulating the spatio-temporal motion of permanent magnets, replicating the experimental setup shown in Fig. S4, using COMSOL Multiphysics. The artificial embryonic node is subjected to zero-velocity (no-slip) boundary conditions on both the walls of the node and the base. The cilia have a zero-velocity condition imposed at their base. A no-slip boundary condition is applied at the cilia-fluid interface. Both the cavity and the surface of the cilia are discretized using three-noded triangular elements.

The cilia are immersed in a viscous fluid and exhibit a Reynolds number (Re) corresponding to the Stokes regime, see supplementary Text S5, affirming the negligible impact of inertial effects. We model the over-damped fluid surrounding the cilia using the Stokes equation:

$$\mu \nabla^2 \boldsymbol{u} = -\nabla p. \tag{3}$$

Here, μ denotes viscosity and ∇p represents the pressure gradient. Simultaneously, the continuity equation $\nabla \cdot \boldsymbol{u} = 0$ describes incompressible flow. Gravitational and thermal effects are neglected. The solutions are derived by applying Green's function in a semi-infinite fluid domain, as provided by (45). The fluid velocity $\boldsymbol{u}^{\mathrm{f}}$ at a point in the fluid $\boldsymbol{r} = (x, y, z)$ due to a point force $\boldsymbol{f}$ exerted on the fluid by the cilia at a position $\boldsymbol{r'}$ is defined as:

$$\boldsymbol{u}^{\mathrm{f}}(\boldsymbol{r}) = \boldsymbol{G}\left(\boldsymbol{r} - \boldsymbol{r}', h(\boldsymbol{r}')\right) \boldsymbol{f}(\boldsymbol{r}'). \tag{4}$$

Here, $h(\boldsymbol{r}')$ signifies the distance of the point force from the bottom of the channel, and

$\boldsymbol{G}\left(\boldsymbol{r}-\boldsymbol{r}', h(\boldsymbol{r}')\right)$ represents the Green's function for a point force $\boldsymbol{f}(\boldsymbol{r}')$ acting in a fluid near a no-slip boundary in Stokes flow. These point forces are distributed over the cilia surface as tractions $\boldsymbol{t}(\boldsymbol{r}')$ such that $\boldsymbol{f}(\boldsymbol{r}') = \int_S \boldsymbol{t}(\boldsymbol{r}')dS$, varying linearly over triangular surface elements. The resulting fluid velocity $\boldsymbol{u}^{\mathrm{f}}(\boldsymbol{r})$ is expressed as a summation and integration over all surface elements (nelm):

$$\boldsymbol{u}^{\mathrm{f}}(\boldsymbol{r}) = \sum_{j=1}^{\mathrm{nelm}} \int_{\mathrm{S}_j} \boldsymbol{G}\left(\boldsymbol{r}-\boldsymbol{r}^j, h(\boldsymbol{r}^j)\right) \boldsymbol{t}(\boldsymbol{r}^j) dS_j. \tag{5}$$

Since this equation holds at every point in the fluid, it can be written for the nodes i on the cilium surface:

$$\boldsymbol{u}^{\mathrm{c}}(\boldsymbol{r}^i) = \sum_{j=1}^{\mathrm{nelm}} \int_{\mathrm{S}_j} \boldsymbol{G}\left(\boldsymbol{r}^i-\boldsymbol{r}^j, h(\boldsymbol{r}^j)\right) \boldsymbol{t}(\boldsymbol{r}^j) dS_j. \tag{6}$$

Equation 6 denotes the velocity of the ith ciliary node due to the traction exerted by the cilia surface on the fluid.

The aforementioned equations are assembled to form a matrix $\boldsymbol{G}$ resulting in $\boldsymbol{u}^c = \boldsymbol{G}\boldsymbol{t}$, which relates the cilia traction on the fluid ($\boldsymbol{t}$) to the cilia velocity ($\boldsymbol{u}^{\mathrm{c}}$). By inverting this relation, the traction exerted by the cilia ($\boldsymbol{t}$) on the fluid can be obtained from the ciliary velocity ($\boldsymbol{u}^{\mathrm{c}}$) that is kinematically prescribed. The traction obtained is used to calculated the fluid velocity ($\boldsymbol{u}^f$) using Eq. 5. To enforce no-slip boundary conditions the top and side walls are discretized by stationary triangular elements and cilia are attached to the bottom wall, coinciding with the semi-infinite no-slip surface defined through the Green's function. To ensure convergence in results, the element size and time-step are carefully selected ensuring both accuracy and computational feasibility in our simulations. It is important to mention that the Green's function becomes singular at the cilia base nodes. To regularize this singularity, the cilia base has been given a small upshift.

Text S7: COMSOL model

We simulate the deflection of primary cilia caused by the simulated flow field using COMSOL Multiphysics v6.1.

We place a primary cilium at the crown cell location as shown in Fig. S17(A). A small control volume is considered around the primary cilia, represented by a box shown in Fig. S17(A). This control volume is modeled as a fluid channel with the primary cilium positioned near the channel inlet with its base fixed. At the channel inlet, a fully developed velocity field derived from the flow simulations of the full node is prescribed, while a zero-pressure condition is applied at the outlet. The remaining walls are subjected to a no-slip boundary condition. The cilium and fluid are discretized using tetrahedral elements.

We use the scaled-down simulated flow field information at the nodes corresponding to the primary cilia using a cubic interpolation scheme (within COMSOL). Subsequently, quasi-static fully-coupled fluid-structure interaction simulations are carried out over a single beating cycle of motile cilia to determine the tip deflection of the primary cilia. Further, using small deflection approximation, we compute the bending angle of primary cilia using its tip displacement. This process is repeated to simulate the tip deflection of primary cilia positioned at various locations within the artificial node.

Text S8: Primary cilia deflection

We derive the tip displacement (w) of a primary cilium subjected to viscous fluid loading using Euler-Bernoulli beam theory (Eq. 7), akin to a cantilever beam.

$$\frac{d^2w}{dy^2} = \frac{M}{EI}, \tag{7}$$

where M is the bending moment, E is the Young's modulus, and I is the second moment of area of the cilium cross-section, see Fig. S17(B). For an infinitesimal segment of length dy along a primary cilium of diameter d, the differential drag force exerted by the surrounding fluid is $dF \propto (ud)dy$, and the corresponding bending moment is $dM \propto ydF \propto (ud)ydy$. The total tip displacement is obtained by double integration of Eq. 7:

$$w \propto \iint \frac{(ud)y}{EI} dydy. \tag{8}$$

For fully developed laminar flow in a rectangular channel, the velocity profile is given by

$$u = 4u_o \left(\frac{y}{h} - \frac{y^2}{h^2} \right),$$

where u_o is the maximum centerline velocity, h is the channel height, and y is measured from the channel base as in Fig. S17(B). Substituting this into Eq. 8 and integration gives

$$w \propto \frac{u_o d}{EI} \left(\frac{l^4}{3h} - \frac{l^5}{5h^2} \right).$$

Expanding I in terms of the cilium diameter ($I \propto d^4$) gives:

$$w \propto \frac{u_o}{Ed^3} \left(\frac{l^4}{3h} - \frac{l^5}{5h^2} \right), \tag{9}$$

showing that the exponent of cilia length lies somewhere between 4 and 5, in accordance with the scaling exponent of 4.4 found in Fig. 4(F) in the main text.

Text S9: Convection-diffusion of particles within the embryonic node

Chemosensing within the node is hypothesized to involve the transport of morphogens (signaling proteins) (3,5,15) or nodal vesicular parcels (NVPs)—morphogen-containing packets (18,26,40)—released into the nodal flow. Their concentration is detected by chemoreceptors that triggers an asymmetric signaling cascade. To investigate chemosensing in our artificial node, we model massless particles that have biologically relevant diffusion coefficients. The particles are considered massless as the flow lies in the low-Reynolds-number regime where viscous forces dominate and inertial effects are negligible, and including particle mass would therefore not change the physics of the particle transport. While signaling particles in biology (morphogens, NVPs, or any other proteins) vary in size and type, we assume a homogeneous population of inert particles, i.e., without bio-chemical affinity.

Particle tracking is performed by initializing particles at predefined locations and simulating their motion using the velocity fields obtained from our flow simulations. Given the periodic nature of ciliary beating, we utilize velocity data from a single beating cycle to iteratively update the particle positions. We focus our analysis on a $20\,\mu\mathrm{m}$-thick rectangular slab in the xz-plane spanning the left of the node to its right. This approach optimizes computational efficiency by limiting the particle distribution analysis in the region of interest rather than the entire node. The slab is discretized into regular 2D-bins to compute particle distributions. Only the particles present within the slab are considered for enumeration at the locations of interest while the particles are continuously allowed to move in and out of the slab. Following the literature (5,18,26, we initially position the particles at the center of the base of the artificial node within a stack of $250\,\mu\mathrm{m}\times250\,\mu\mathrm{m}\times25\,\mu\mathrm{m}$, see Fig. 5(A).

Previous studies (5,15,18,40) suggest that morphogens are primarily sensed at the bottom surface of the node, while NVPs have been suggested to rupture (releasing morphogens) near

perinodal (immotile) cilia or nodal crown cells at the bottom near the node walls (18,40). In the context of mechanosensing, Katoh et al. (29) speculated on the asymmetric localization of channel proteins (Hv1) and structural proteins (LRRCC1) at the left and right immotile cilia that might play a role in the breaking of L-R symmetry. Based on these studies, we track the particle accumulation at four key sensing sites: the left and right central and bottom receptor sites as shown in Fig. 5(A) and Fig. S20(A).

On top of convection, also diffusion plays a key role at these dimensions. To model its contribution, we add random Brownian motion to the convective motion of each particle. The Brownian motion is modeled using the Stokes-Einstein relation (40,41). The updated particle positions are computed by summing the convective displacement and a random diffusion component drawn from a normal distribution with zero mean and σ^2 variance according to:

$$\boldsymbol{x}_t^i = \boldsymbol{x}_{t-1}^i + \boldsymbol{u}_t^i dt + \delta \boldsymbol{v}_t, \tag{10}$$

where $\boldsymbol{x}_t^i$ and $\boldsymbol{x}_{t-1}^i$ denote the position vectors of the ith particle at times t and $t-1$, respectively, $\boldsymbol{u}_t^i$ is the velocity vector of the ith particle at time t as obtained from our flow simulations and $\delta \boldsymbol{v}_t$ is a diffusional step taken from a three-dimensional normal distribution with zero mean and $2Ddt$ variance. Here, D is the diffusion coefficient and dt is the time-step.

For high diffusion coefficients, large displacements occasionally cause the particles to exit the artificial node. In such conditions, we reposition them near the node wall, re-evaluate their velocity, and iteratively adjust their displacement until they remain within the node.

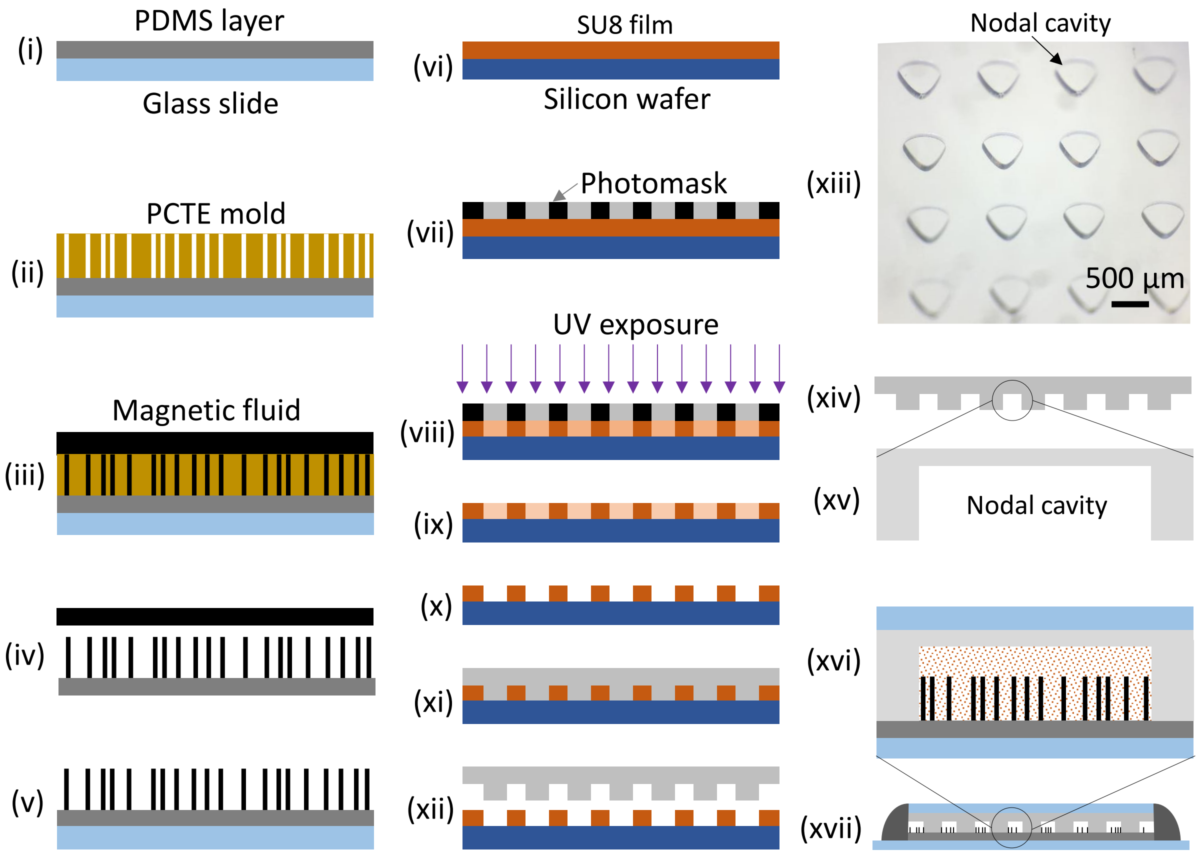

Fig. S1: Fabrication process: (I) PDMS coated glass slide. (ii) PCTE mold on cured PDMS layer. (iii) Addition of magnetic fluid to the mold. (iv) Detachment of top magnetic layer. (v) Released cilia on the glass substrate. (vi) Spin-coated wafer before and after baking. (vii) Placing photomask on the baked SU-8. (viii) UV exposure of the SU-8 layer. (ix) Photomask removal followed by post-baking. (x) Developed wafer with SU-8 template. (xi-xii) PDMS molding and demolding. (xiii) Stereo-microscopic image of demolded triangular nodal cavities. (xiv) side view of nodal cavities. (xv) Zoomed view. (xvi) Integrated embryonic node. (xvii) Integrated device and with ends sealed.

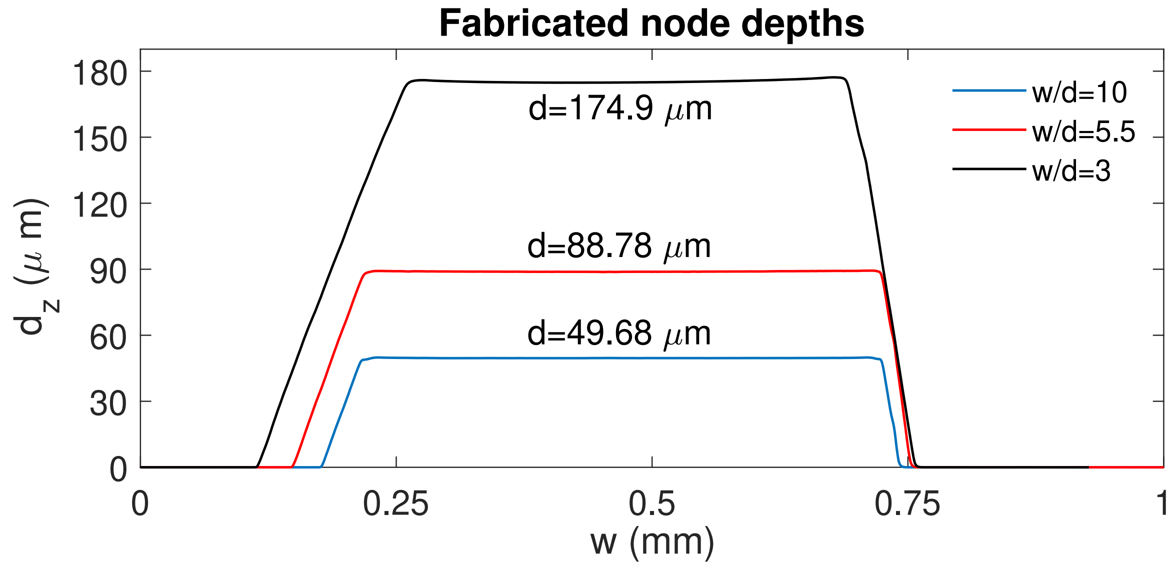

Fig. S2: Nodal depth d measured using a stylus profilometer (DektakXT from BRUKER) across the width ($w = 500\,\mu\mathrm{m}$) of the nodes. Multiple measurements indicate depths of 170 ± 5, 90 ± 2, and $50 \pm 1\,\mu\mathrm{m}$ for nodes with $w/d = 3,\ 5.5$, and 10 respectively. The slanted sides of the nodes seen here are a result of the angled profile of the stylus generating an inclined line even though the walls of the node are straight.

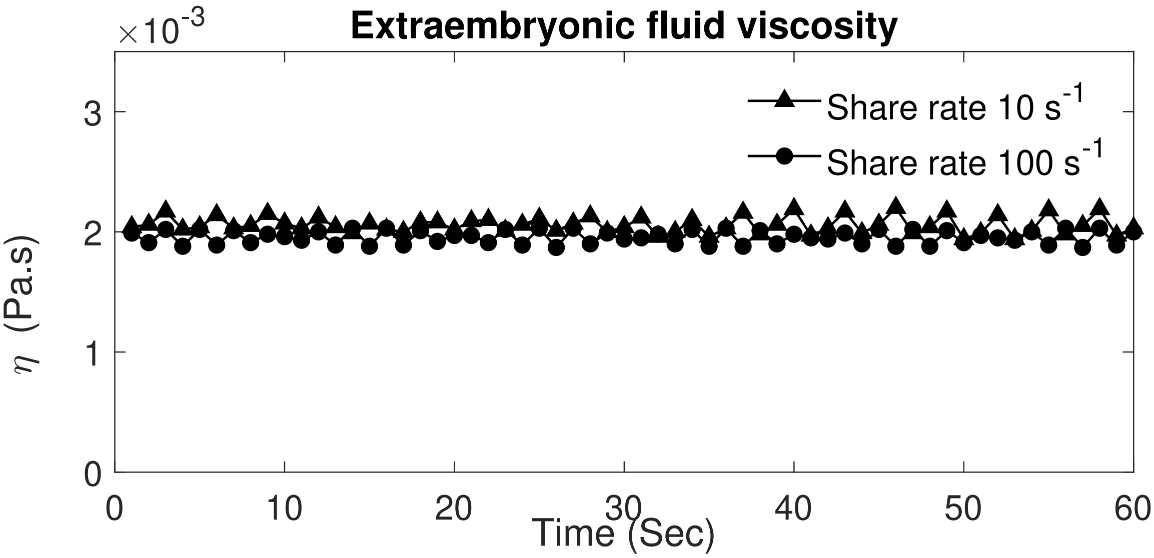

Fig. S3: Fluid viscosity measured in a Couette geometry using Physica MCR 501 rheometer from Anton-Paar. Identical viscosity measurements obtained at two different share rates show the Newtonian nature of the prepared solution representing the extraembryonic fluid. The measurements are done without the tracer particles added in.

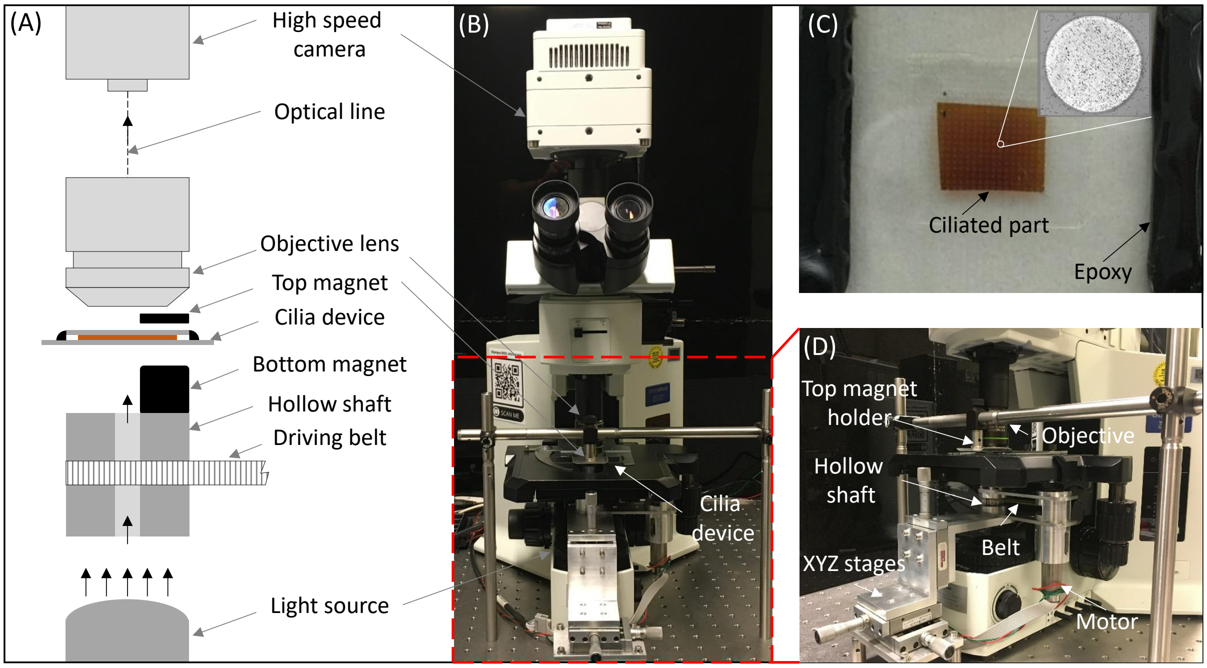

Fig. S4:(A) Schematic diagram showing the position of different parts of the integrated setup. The bottom magnet is placed on a rotating hollow shaft driven by a motor attached through a belt-pulley mechanism. The hollow shaft allows the light-source to be placed below it and the bottom magnet near to the cilia device above it. The top magnet is placed between the microscope objective-lens and the cilia device where the lens has a working distance larger than the thickness of the top magnet. The motor driving the hollow shaft and the high-speed camera attached at the top of the microscope are connected to a PC for magnetic actuation control and recordings. (B) Image of the setup showing different components. (c) Image of the cilia device showing the ciliated part and the nodal cavities made from transparent PDMS integrated between two glass slides, one shorter than the other, and held in place by the epoxy sealing. The zoomed-up inset image shows a circular artificial embryonic node of diameter $500\ \mu\mathrm{m}$ (D) Close-up view of the magnetic actuator mounted on a combined XYZ-stage for its precise positioning with respect to the cilia device and the microscope optical axis.

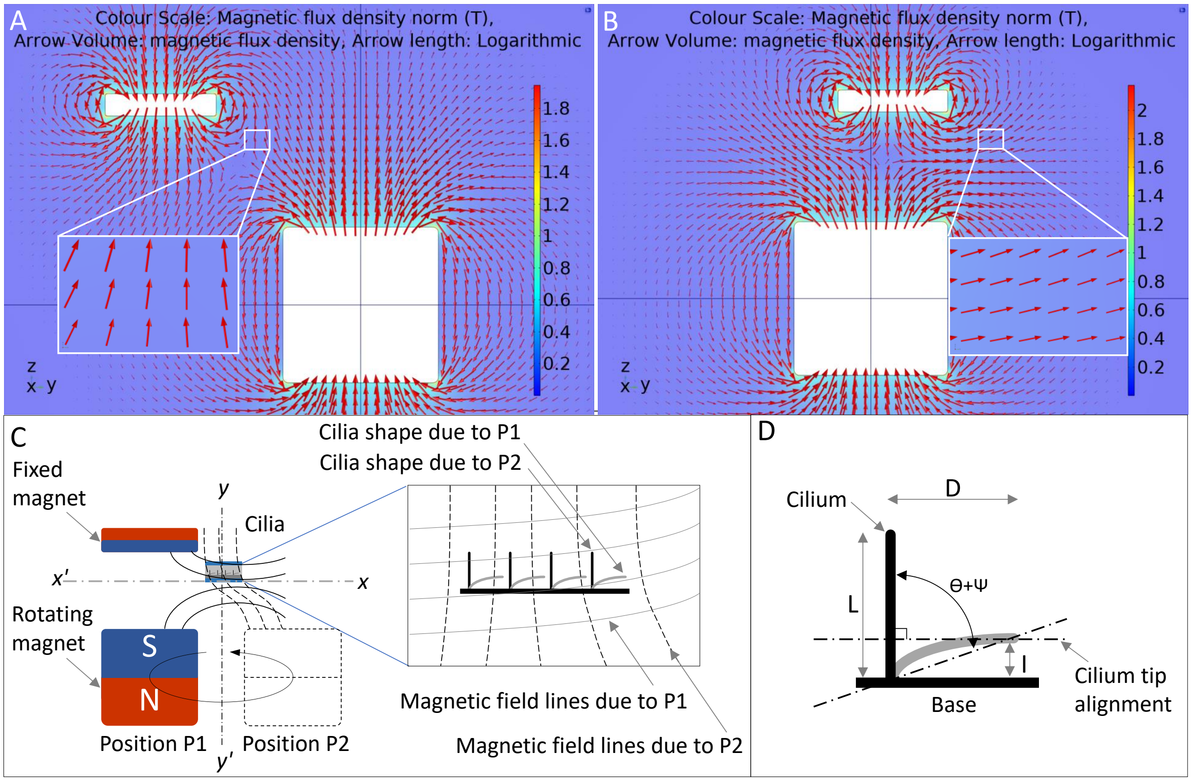

Fig. S5: Tilted conical motion induced by the magnetic field: (A) A stationary COMSOL simulation of the two-magnet arrangement generating a vertical field at the position of the cilia. A $7 \times 7 \times 7\,mm$ magnet is used on the bottom side and a $5 \times 5 \times 1\,mm$ magnet is used on the top side. The two magnets are $5 - 6\,mm$ apart vertically and $3\,mm$ horizontally. The simulation results are confirmed by measuring the field strength, using a Gauss meter (by FW Bell), of the $7\ \mathrm{mm}^3$ magnet along its central axis and compared with the simulations separately. (B) Horizontal field generated by the magnets when the bottom magnet rotates and positions below the top magnet; the cilia tend to align with the magnetic field, and therefore, during one rotation of the lower magnetic the cilia orientation changes from vertical to horizontal while rotating, producing the TCM. (C) Schematic representation of the magnets and the filed lines around the cilia patch. The cilia are placed at the origin of the xx' and yy' axes shown here which are $1.5\,mm$ from the bottom face of the top magnet $1.5\,mm$ from the front face of the magnets respectively. The cilia shape in the two extreme positions is sketched. (D) A cilium with both the vertical and the horizontal/ deflected alignment is shown here. At its maximum deflection the cilium tip is nearly parallel to the substrate and at a distance I from it. Measuring the top-view projection (D) of the deflected cilium, the limits of the tilt angle $(\theta + \psi)$ are calculated by assuming the cilium may either rotate/ deflect purely about its base or bend 90^0 at some point L_x so that $L_x + D = L$ (i.e. the cilium length). With an average value of $D = 13.5\ \mu\mathrm{m}$ and $L = 22\ \mu\mathrm{m}$, measured using ImageJ, the maximum and minimum values of the tilt angle are 60^0 and 40^0 respectively. The actual deflection lies between these two values and is taken as 50^0.

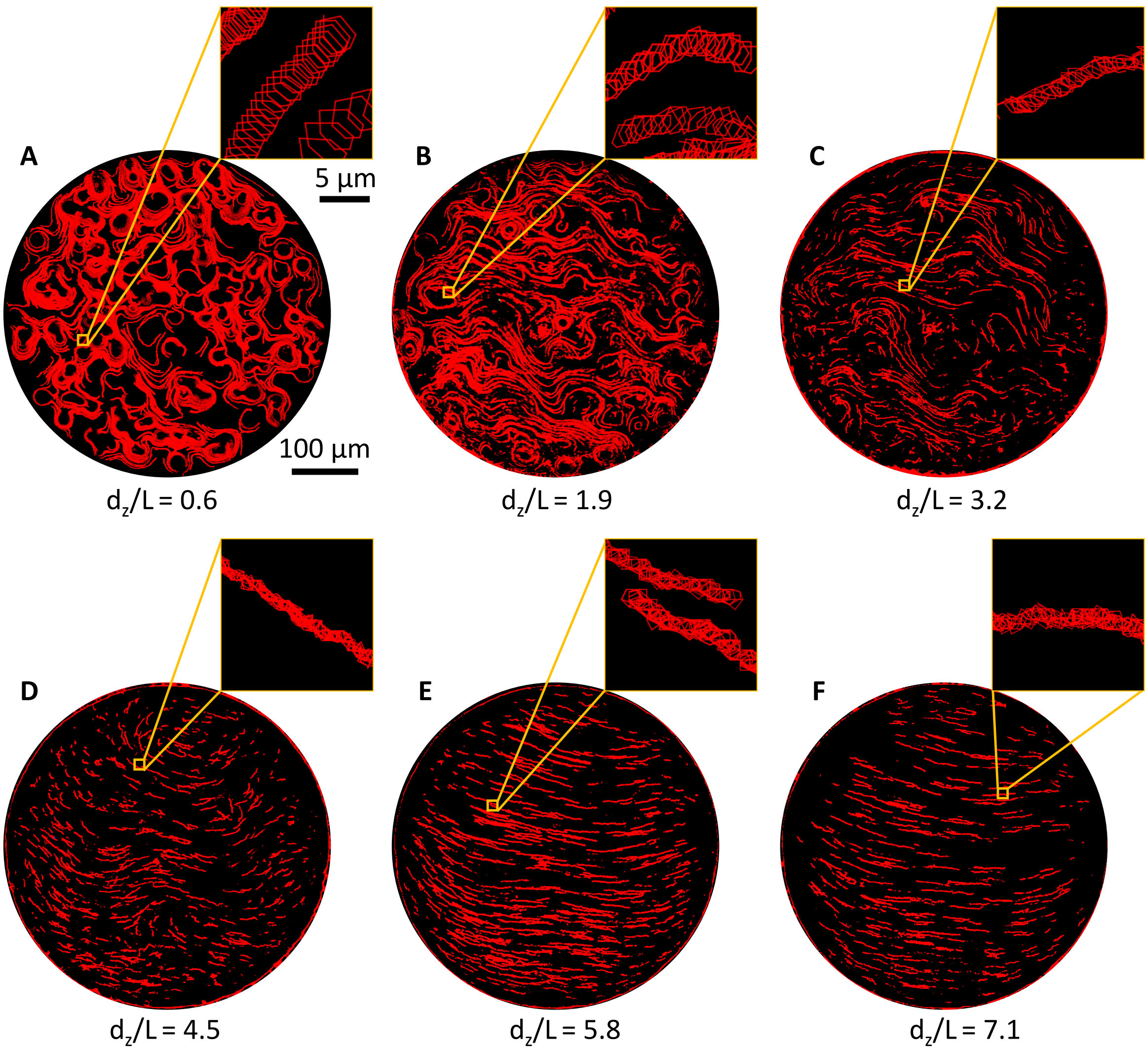

Fig. S6: Loopy motion of the nodal flow: (A-F) Tracing of particles, captured at a frame rate of 60 fps, is performed using TrackMate plugin (46) of the open source software 'FIJI ImageJ'. The particles are traced across six equidistant layers spanning the depth of the artificial embryonic node with $w/d = 3$. Each sub-figure shows a magnified view of tracks in the corresponding layer. The zoomed-in views of tracks in A, B, and C show a spiral/ loopy motion of the particles in the bottom half of the node. The faceted shape of the loops is due to a finite number of frames $(60 \text{ fps}/10 \text{ Hz} = 6)$ capturing each loop produced by a single cycle of cilia rotation, see supplementary Movie S3. In contrast, the loops in D, E, and F have smaller amplitudes, indicating a reduction in fluid motion in the upper half of the node. The tracks in these layers are overlapped due to the smaller amplitude of the net forward motion, see supplementary Movie S4. Particles in each layer are traced over a time period of 30s, with the cilia actuated at a frequency of $\omega = 10$ Hz. Scale bars in 'A' apply to all the figures, A-F.

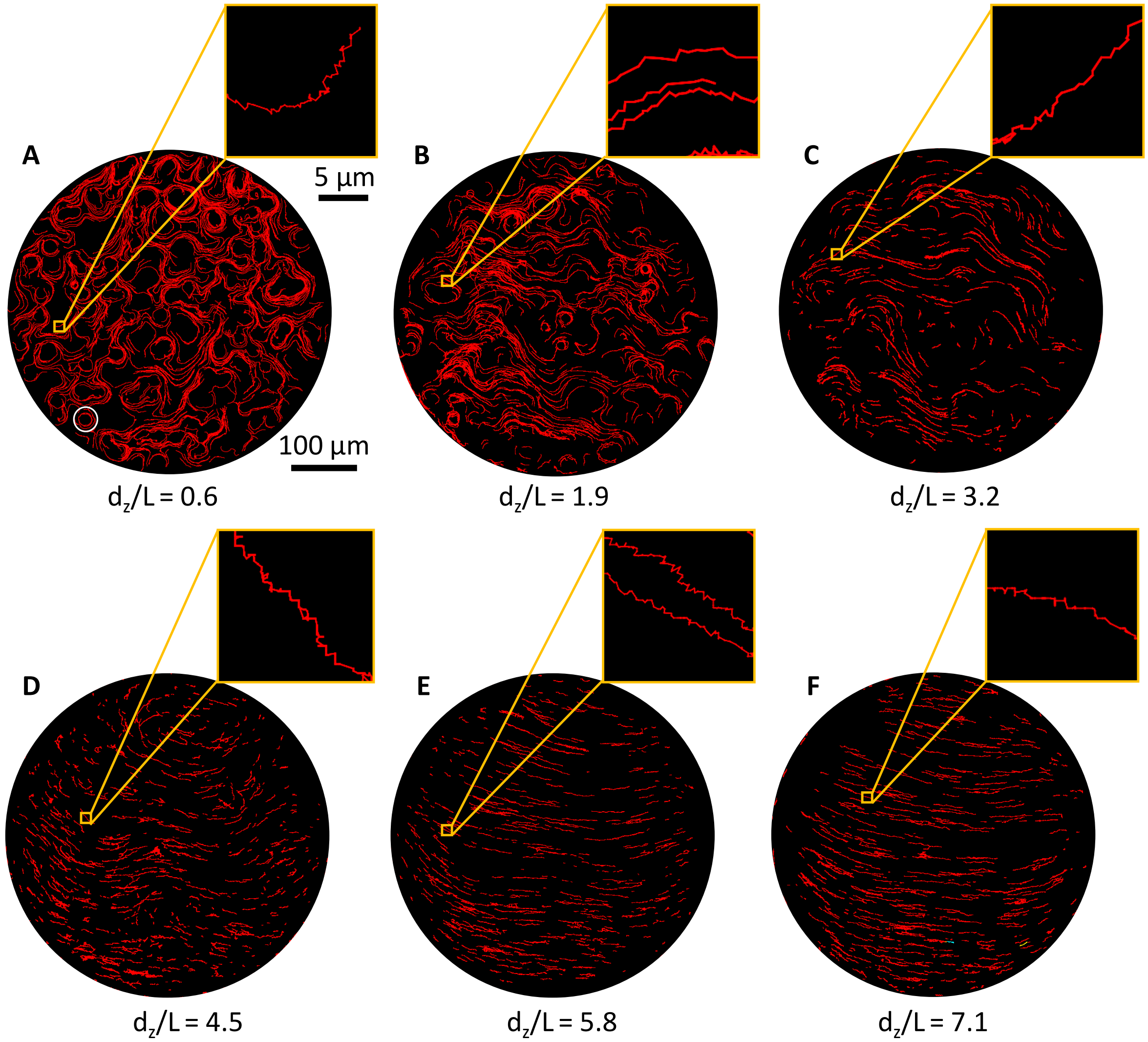

Fig. S7: Steady-state nodal flow: (A-F) The net displacement of particles at the end of each cilia rotation is isolated by matching the particle-capture frame rate to the cilia actuation frequency of 10 Hz thereby eliminating the loopy motion in the fluid shown in Fig. S6. For comparison, all the traced layers shown in Supplementary Fig. S6 have been reanalyzed here after reducing the frame rate from 60 to 10 fps to capture the net fluid motion. In the bottom-most layer with $d_z/L = 0.6$ in 'A', the circular tracks, white circle, indicate the rotating cilia positions as the particles trapped within the cilia vortices undergo a circular motion. Differences in the circular-track sizes arise due to the absence of particles within some of the cilia vortices. Closely spaced cilia are identified by non-circular bounded tracks around them. All the zoomed-in images, A-F, show the net displacement tracks followed by the particles. Particles in each layer are traced over a time period of 30s, with the cilia actuated at a frequency of $\omega = 10$ Hz. Scale bars in 'A' apply to all the figures, A-F.

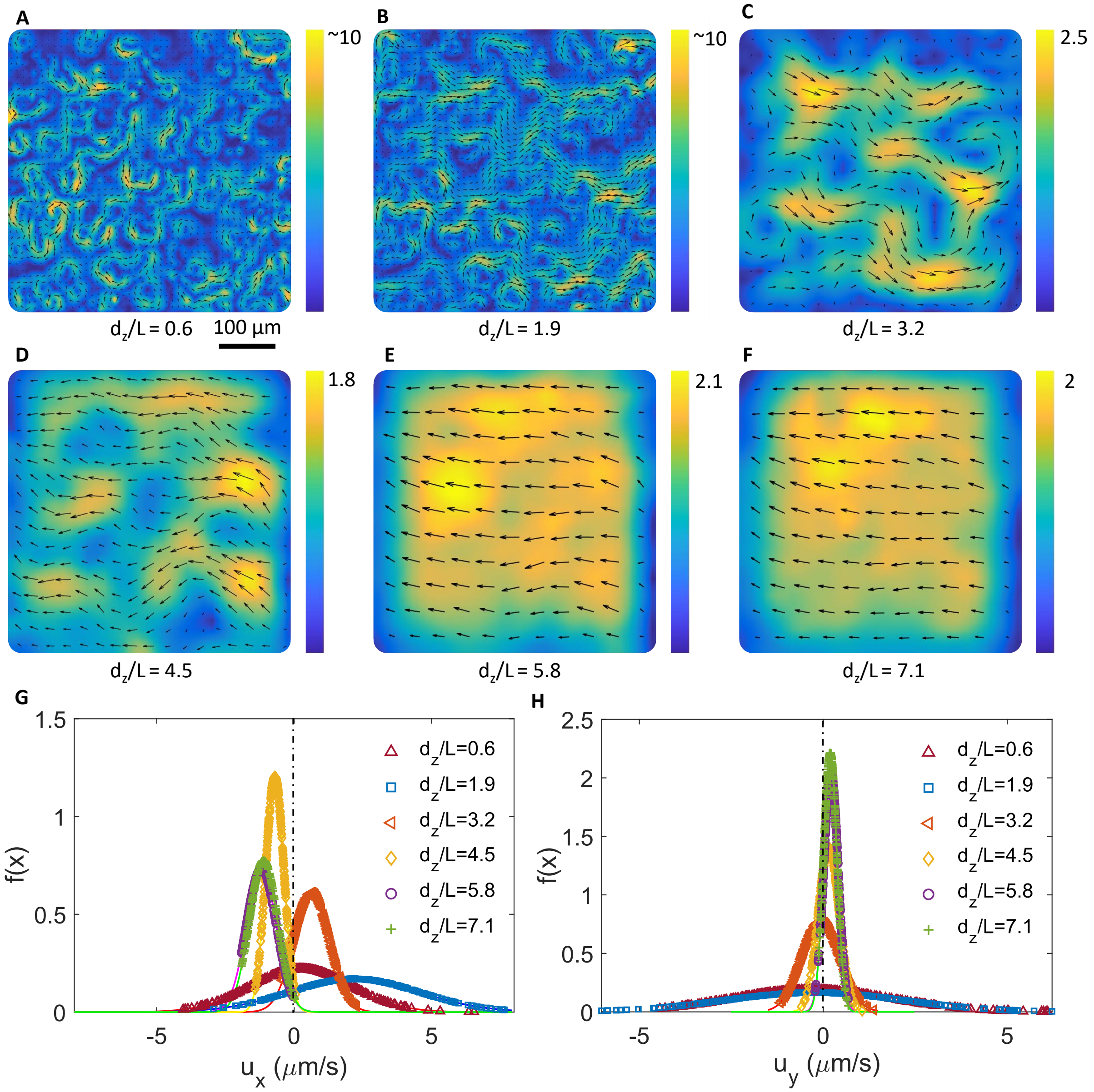

Fig. S8: Square artificial embryonic node with $w/d = 3$, $N_c = 187$ and with cilia actuated at $\omega = 10$ Hz. (A-F) Multidirectional flow, in lower layers, and its evolution first into a multidirectional net leftward flow in the middle layers and then into a return unidirectional flow in the upper part of the node, as observed in the circular node with $w/d = 3$ in Fig. 2, also shown in Movie S6. (G) Distribution of u_x showing extremely weak or no net flow below the cilia tip $(d_z/L = 0.6)$ and a net leftward flow above the cilia tip till half the nodal depth where $d_z/L = 3.2$. All velocity points almost entirely in the right half of the graph for layers with $d_z/L = 4.5 - 7.1$ show the rightward flow in the upper part of node like in the circular node. (H) Symmetric distribution of u_y around the y-axis is identical to the circular node u_y distribution.

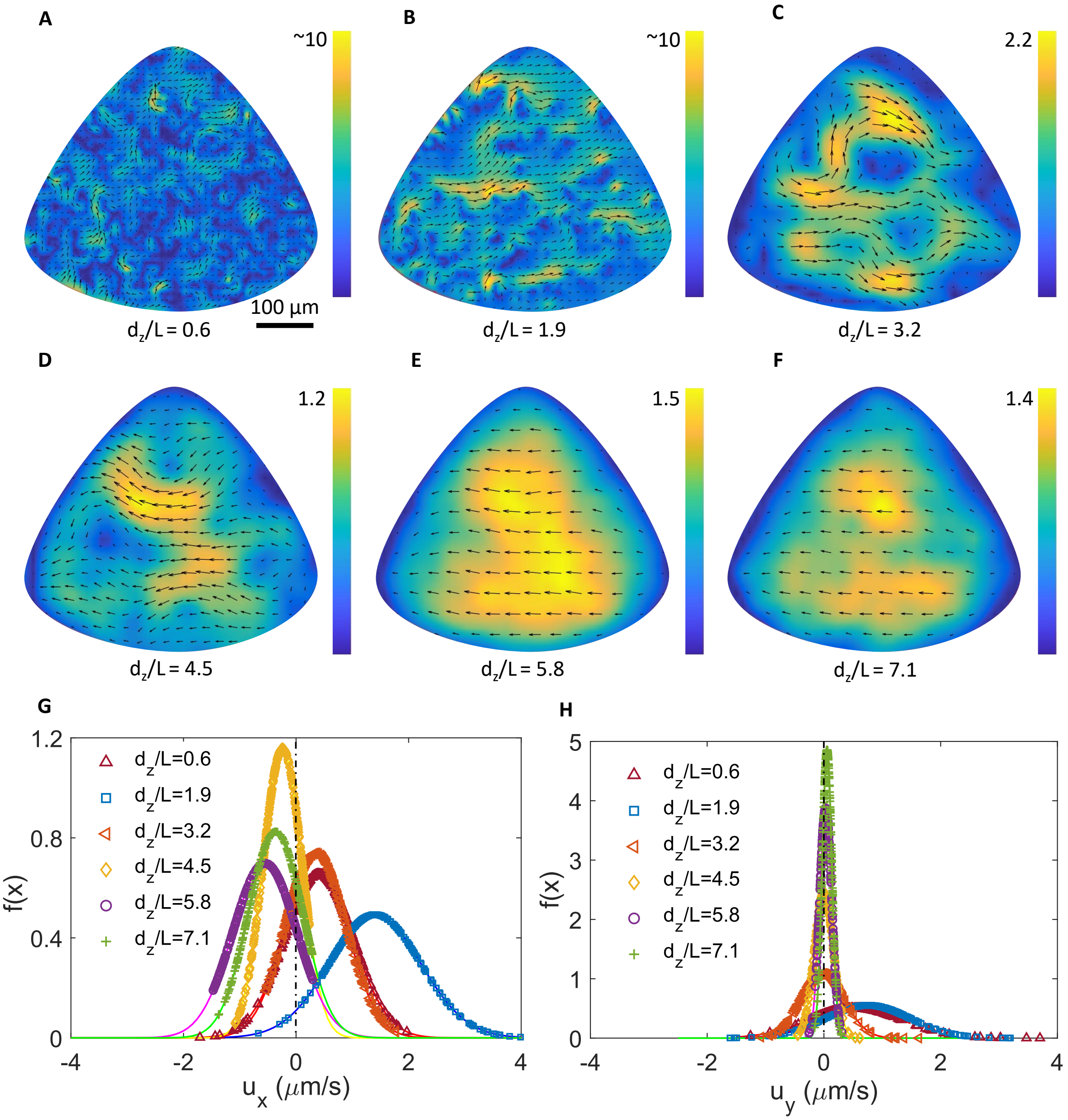

Fig. S9: Triangular artificial embryonic node with $w/d = 3$, $N_c = 120$ and with cilia actuated at $\omega = 10$ Hz. (B-G) Flow evolution, from bottom to top of the node, from multidirectional to directional with flow reversal, consistent with the flow seen in circular and square nodes with $w/d = 3$ (as shown in Fig. 2 and Fig. S8, also shown in Movie S7). (I-J) Velocity component distributions at different depths for the triangular nodes with $w/d = 3$.

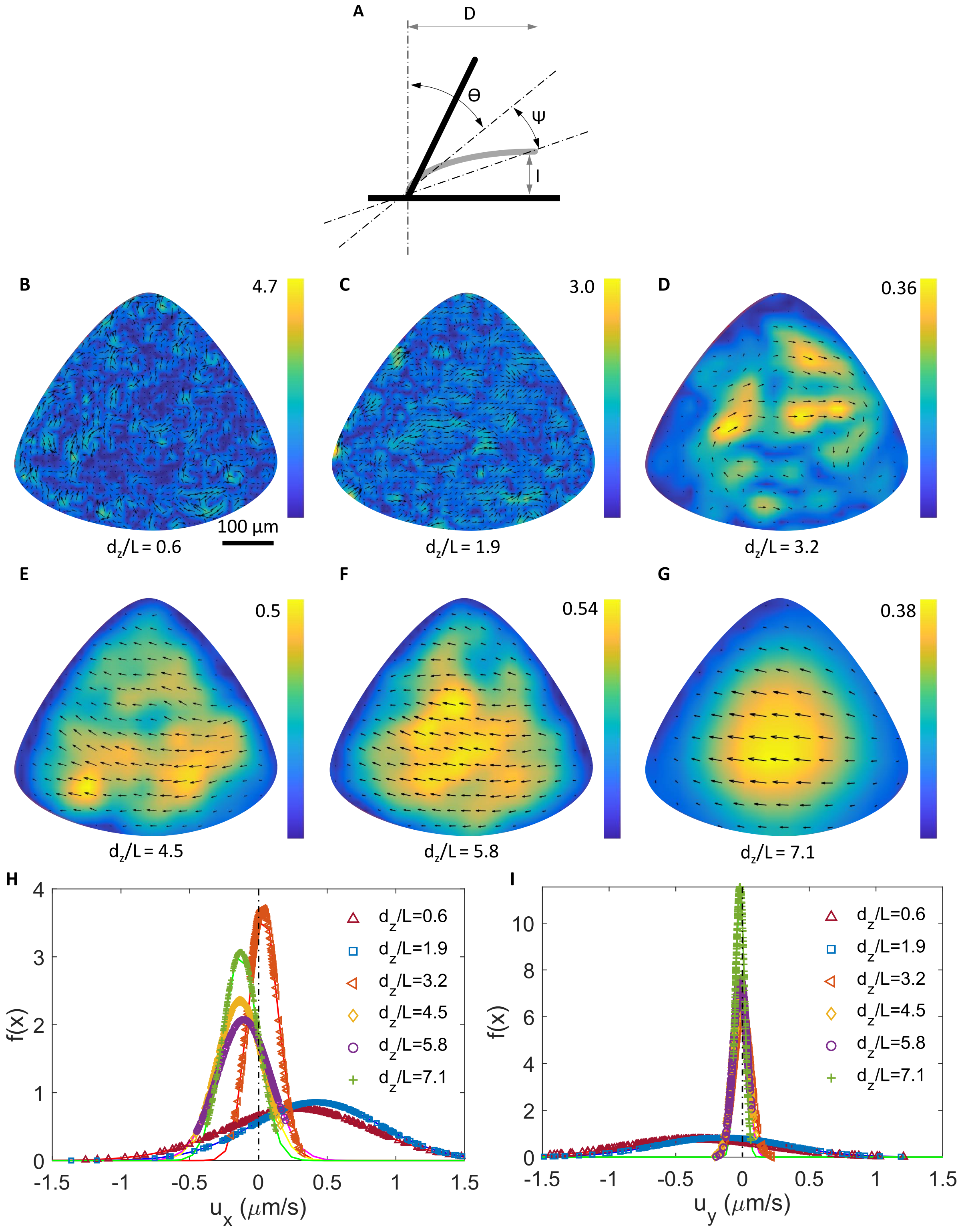

Fig. S10: Caption on the next page.

Fig.S10: (A) Schematic representation of a cilium to exhibiting TCM with the tilt angles θ and ψ adjusted between their maximum and minimum limits to examine the nodal flow generated by a less effective TCM. Measuring the average cilium projection (D) at its minimum and maximum bending equal to $7.5\ \mu\mathrm{m}$ and $13.5\ \mu\mathrm{m}$ respectively, the tilt angles take an average value of $\theta = 36.5^0$ and $\psi = 13.5^0$. At these angles the cilia rotate near the surface and never retract back to a fully vertical orientation. (B-D) Multidirectional flow observed in the lower part of a triangular node with $w/d = 3$, with cilia rotating at $10\ \mathrm{Hz}$ with the TCM angles mentioned above. (E-G) Directional return flow occurs in the upper part of the node. The flow generated by the less effective TCM is comparable with the nodal flow developed due to the most efficient TCM angles where $\theta = \psi = 25^0$, and shown in Fig. 2 and Fig. S9. The corresponding cilia motion and fluid flow are shown in Movie S8.

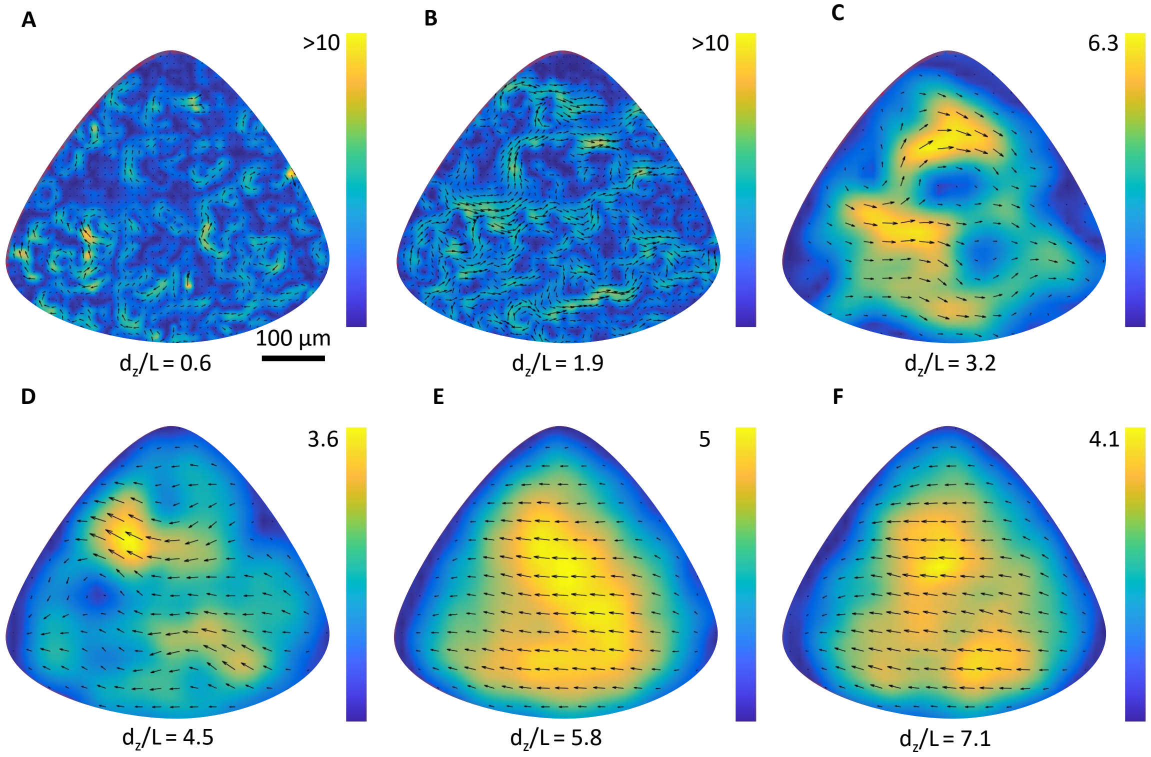

Fig. S11: Velocity distribution plots of a nodal flow measured in a triangular node with $w/d = 3$ with the cilia actuation frequency 5 times higher than the typical nodal frequency of $10\,\mathrm{Hz}$. The distribution is similar to that found at $\omega = 10\ \mathrm{Hz}$ (see Fig. S9).

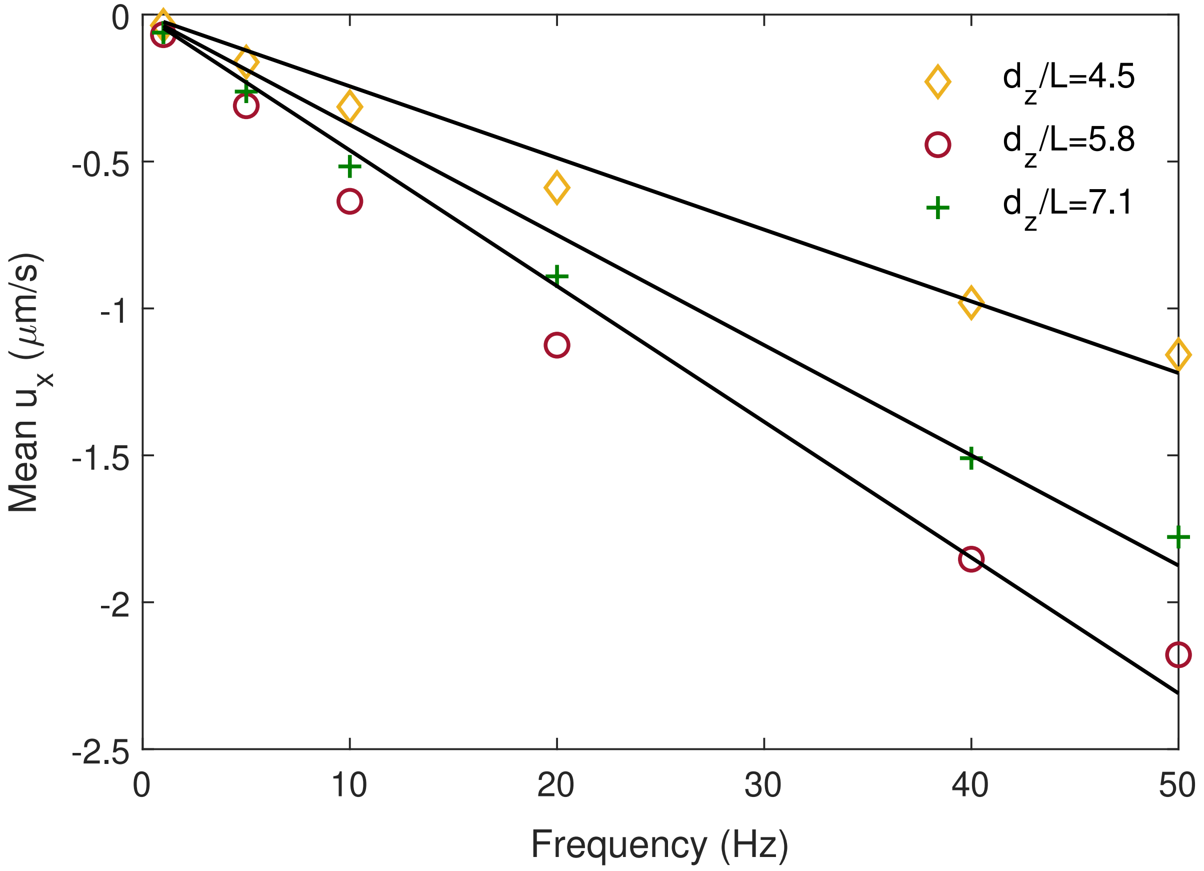

Fig. S12: Linearly increasing magnitude of mean flow velocities with actuation frequency ω at different nodal depths indicate a conserved nodal flow. Supplementary Movie S9 compares the nodal flow at minimum and maximum cilia actuation frequency.

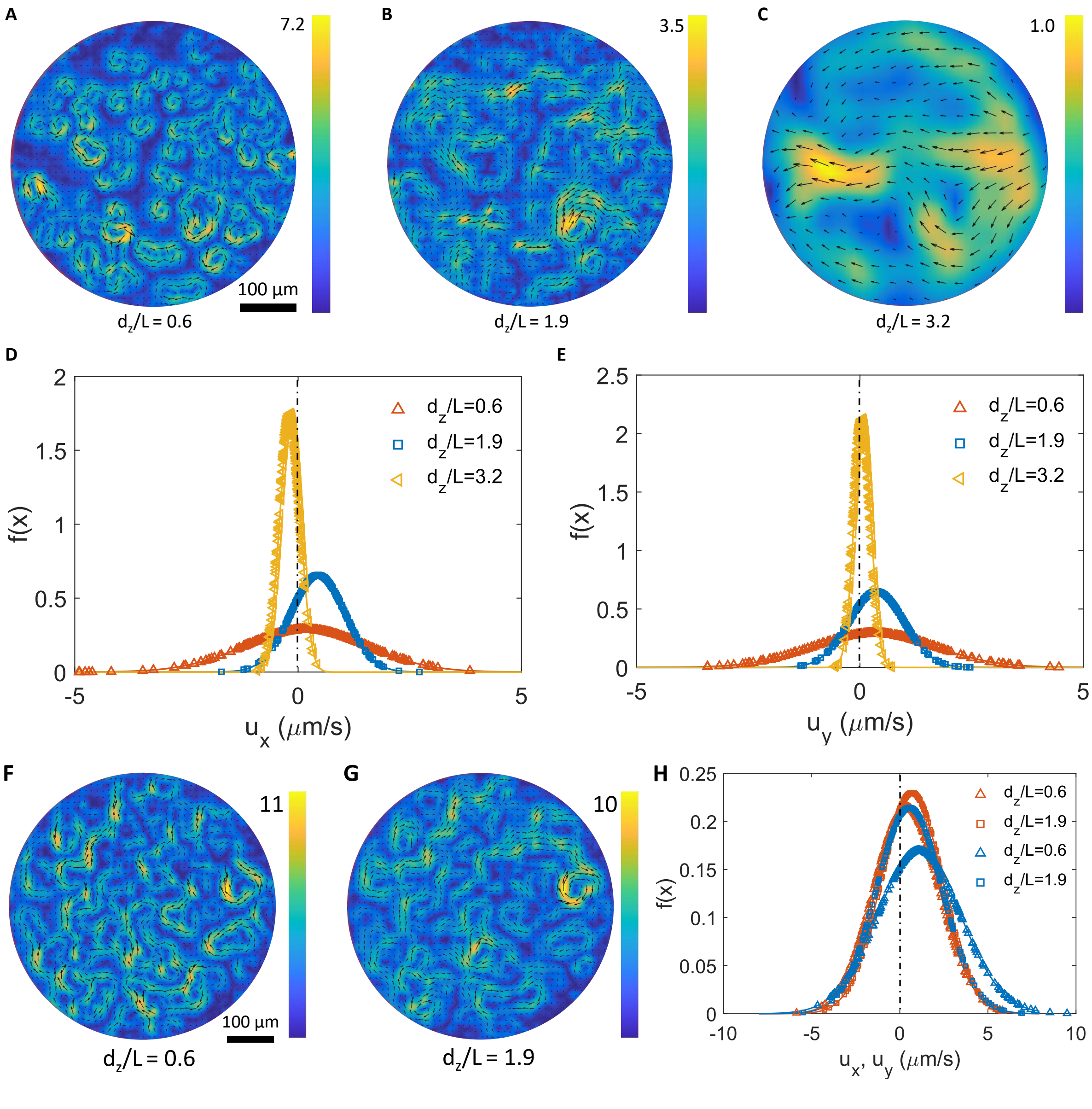

Fig. S13: Caption on the next page.

Fig.S13: Nodal flow characterization in circular artificial embryonic nodes with shallow depth, i.e. aspect ratio $w/d = 5.5$ ($w = h = 500\mu$m and $d = 90\mu$m) and $N_c = 127$ in (A-E) and aspect ratio $w/d = 10$ ($w = 500\,\mu$m and $d = 50\,\mu$m) and $N_c = 140$ in (F-H); in both cases, $\omega = 10\,$Hz: (A-B) Colour and velocity maps based on PIV analysis show multidirectional velocity patterns in the node, gradually coarsening from the layer below the cilia tips (at $d_z = 15\ \mu$m or $d_z/L = 0.6$) to the layer above the tips approximately at the node centre (at $d_z = 40\mu$m or $d_z/L = 1.9$)), which is similar to the patterns in the lower layers seen in the deeper node (Fig. 2(D-E)) (C) The flow patterns in the upper layer close to the top of the node (at $d_z = 75\mu$m or $d_z/L = 3.2$)) show differences with those seen in the deeper node (Fig. 2(H-I)), being multidirectional and much less uniform, but most velocity vectors point towards the nodal right representing the return flow in the node. (D) Normal distribution functions of the velocity component show a weak leftward flow (positive) in the layer just above the cilia tips at $d_z = 1.9$ and a weak net return flow to the right (positive) in the layer close to the top of the node at $d_z = 3.2$. (E) Normal distribution functions of the velocity component u_y are approximately symmetric around the origin throughout the nodal depth, indicating that there is no net flow in the y-direction of the node. Movie S10 shows the corresponding nodal flow. The results for square and triangular nodes with the same w/d are very similar, as shown in the figures below. (F-G) In the node with $w/d = 10$, multidirectional flow patterns are observed throughout the depth of the node, measured in layers at $d_z = 15\ \mu$m or $d_z/L = 0.6$ and $d_z = 40\ \mu$m or $d_z/L = 1.9$. (H) The normal distribution functions of both velocity components u_x (red) and u_y (blue) are symmetric around the origin for both layers, indicating that there is no net directional flow. Movie S11 shows the corresponding nodal flow. The results for circular and square nodes with the same w/d are very similar, as shown in the figures below.

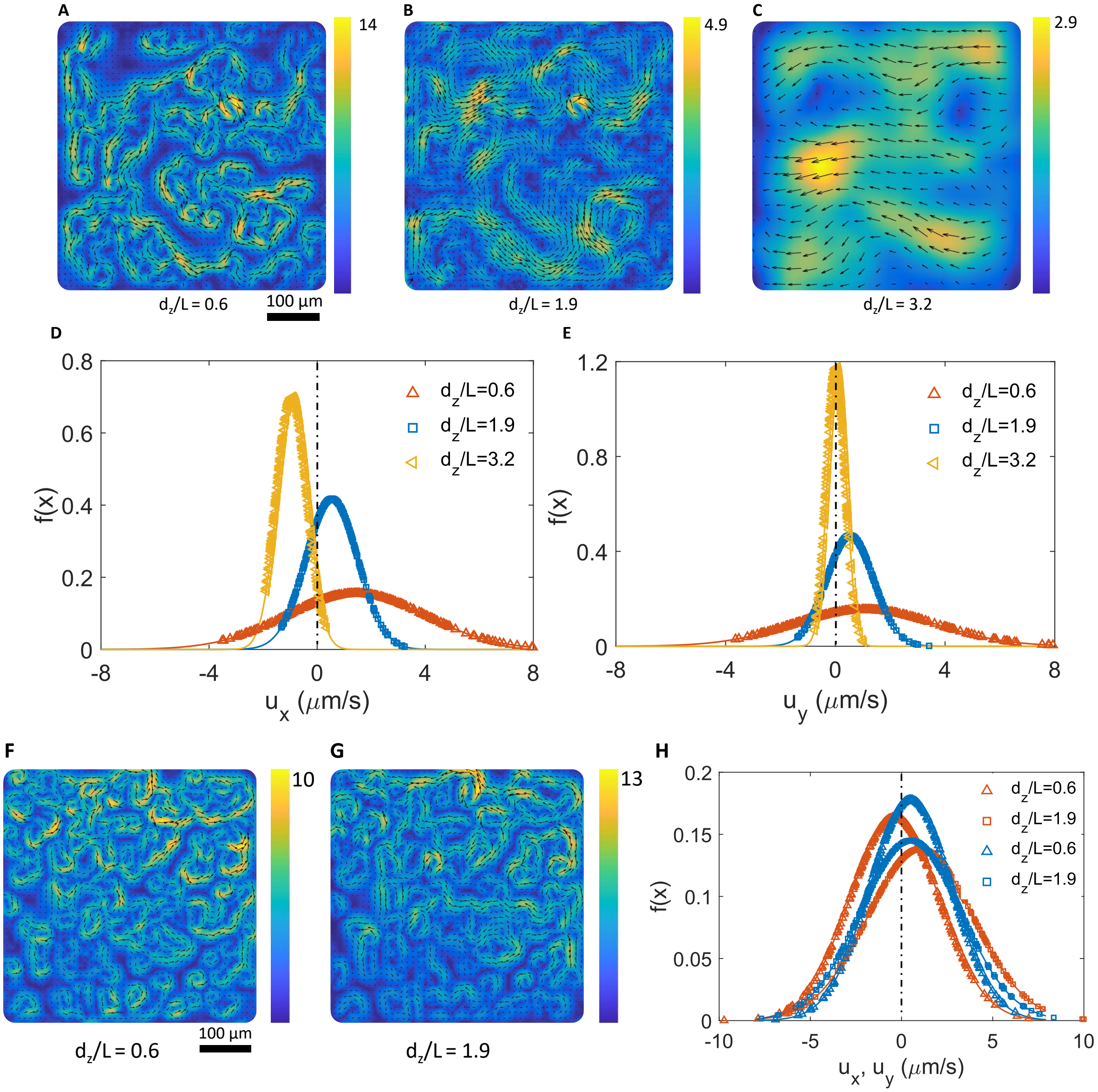

Fig. S14: Nodal flow characterization in square artificial embryonic nodes with shallow depth, i.e. aspect ratio $w/d = 5.5$ ($w = 500\ \mu$m and $d = 90\ \mu$m) and $N_c = 178$ in (A-E) and aspect ratio $w/d = 10$ ($w = 500\ \mu$m and $d = 50\ \mu$m) and $N_c = 215$ in (F-H); in both cases, $\omega = 10$ Hz: (A-B) The nodal flow in this node shows a multidirectional flow throughout the nodal depth with a predominantly rightward flow in the upper part, also shown in Movie S12. (D-E) Velocity distributions for the square node with $w/d = 5.5$. The results are similar to those of the circular node with $w/d = 5.5$, see Fig. S13(A-E). (F-G) The multidirectional nodal flow in this node is similar to that observed in the shallow circular node with $w/d = 10$, also shown in Movie S13. (H) Symmetric velocity distributions indicate no net flow in x or y direction. The results are similar to that of the circular nodes with $w/d = 10$ (see Fig. S13(F-I)).

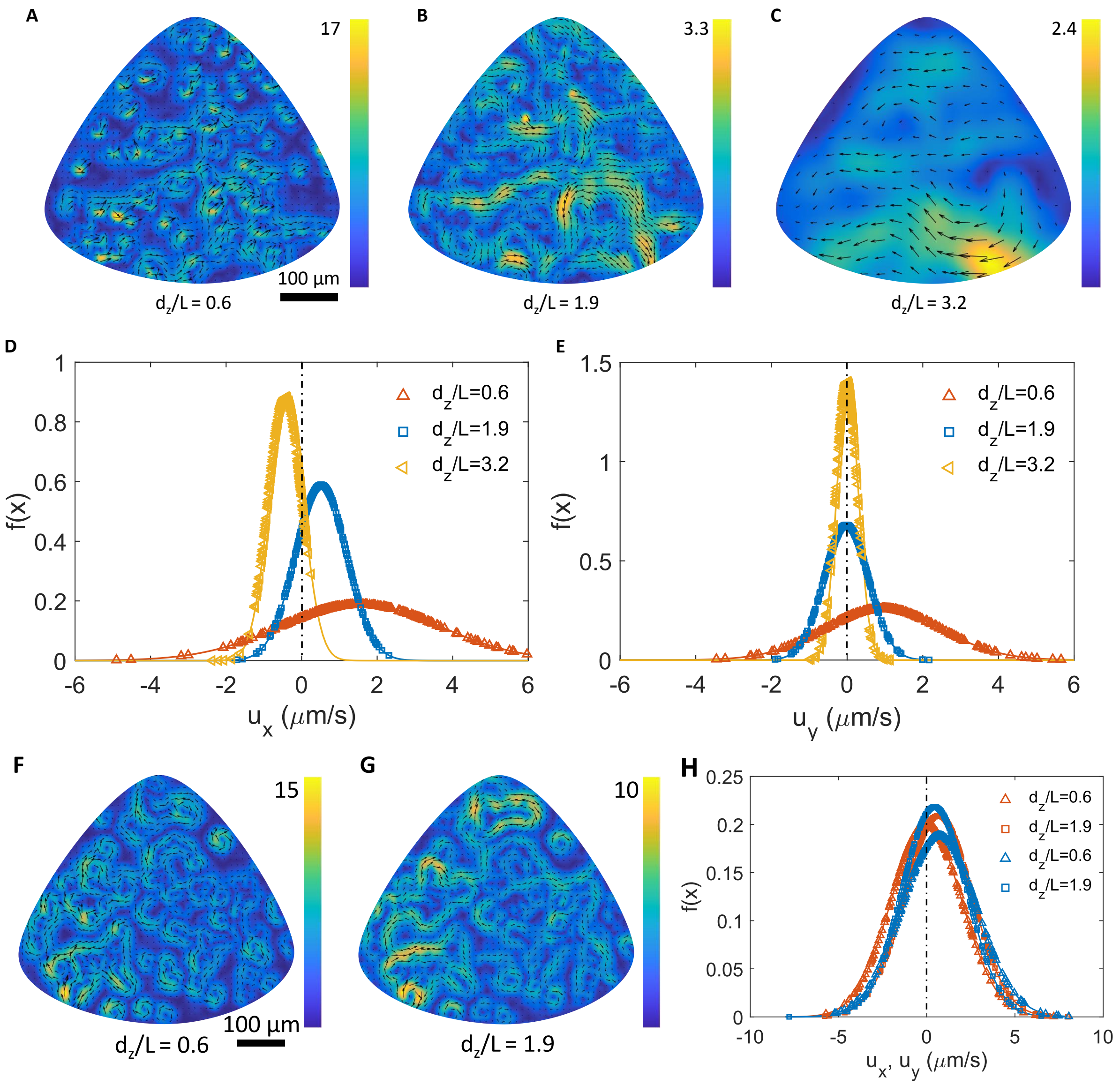

Fig. S15: Triangular node with $w/d = 5.5$ and 10 and $N_c = 109$ and 120, respectively. (A-C) The nodal flow in this node at $\omega = 10$ Hz shows a multidirectional flow throughout the nodal depth with a net leftward flow in the lower part and a predominantly rightward flow in the upper part at $d_z/L = 3.2$, also shown in Movie S14. (D-E) Flow velocity distributions for the triangular node with $w/d = 5.5$. The results are similar to those of the circular and square node with $w/d = 5.5$ (Fig. S13(A-E) and Fig.S14(A-E)). (F-G) A shallow triangular node with $w/d = 10$. A multidirectional flow pattern is generated in the node throughout its depth, also shown in Movie S15. (H) Symmetric velocity distributions indicate no net flow in x or y direction. The results are similar to those of the circular and square node with $w/d = 10$ (see Fig. S13(F-H) and and Fig.S14(F-H)), showing the independence of nodal flow on its shape.

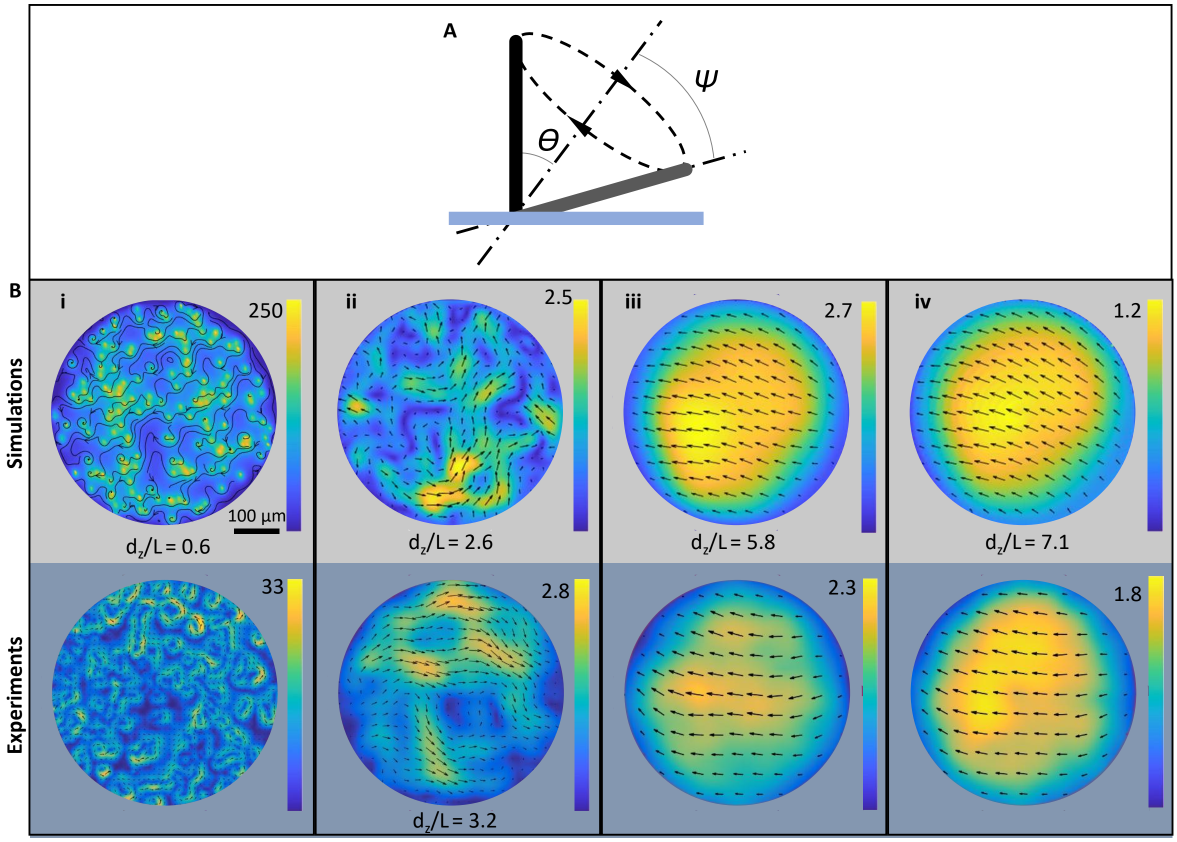

Fig. S16: (A) The rigid body cilium considered in the simulated model undergoing TCM by rotating about its base, see Movie S16. The tilt angle $\theta + \psi$ is deduced from the magnetic field direction variation over one complete rotation of the bottom magnetic in the two-magnetic configuration setup shown in Fig. S5. (B) Simulated and PIV analysis of nodal flow at different depths (d_z/L) of the artificial embryonic node. (i) Fluid flow below the cilia tips at $d_z/\mathrm{L} = 0.6$ shows multidirectional flow with circulations around the cilia; seen in the streamlines of the simulated flow and the arrow arrangement of the PIV amp. Low velocities at the cilia tips seen in the PIV map are due to the impossibility of having a particle attached to the cilia tips representing the fluid flow. (ii) Coarsened fluid flow above the cilia and just below the returning flow in the node with a similar degree of flow coarsening. Different depths (d_z/L) are compared here as the flow returns at a slightly lower depth in the simulated node. Given the difference in cilia shapes used in the simulations compared to those observed in experiments, the flow characteristics below the transition line are dissimilar, although both exhibit a net leftward flow. (iii-iv) Unidirectional flow developed in the upper part of the node is consistent with the measured flow (PIV). The maximum velocities are also comparable in all the layers above the cilia tips, ‘ii-iv’.

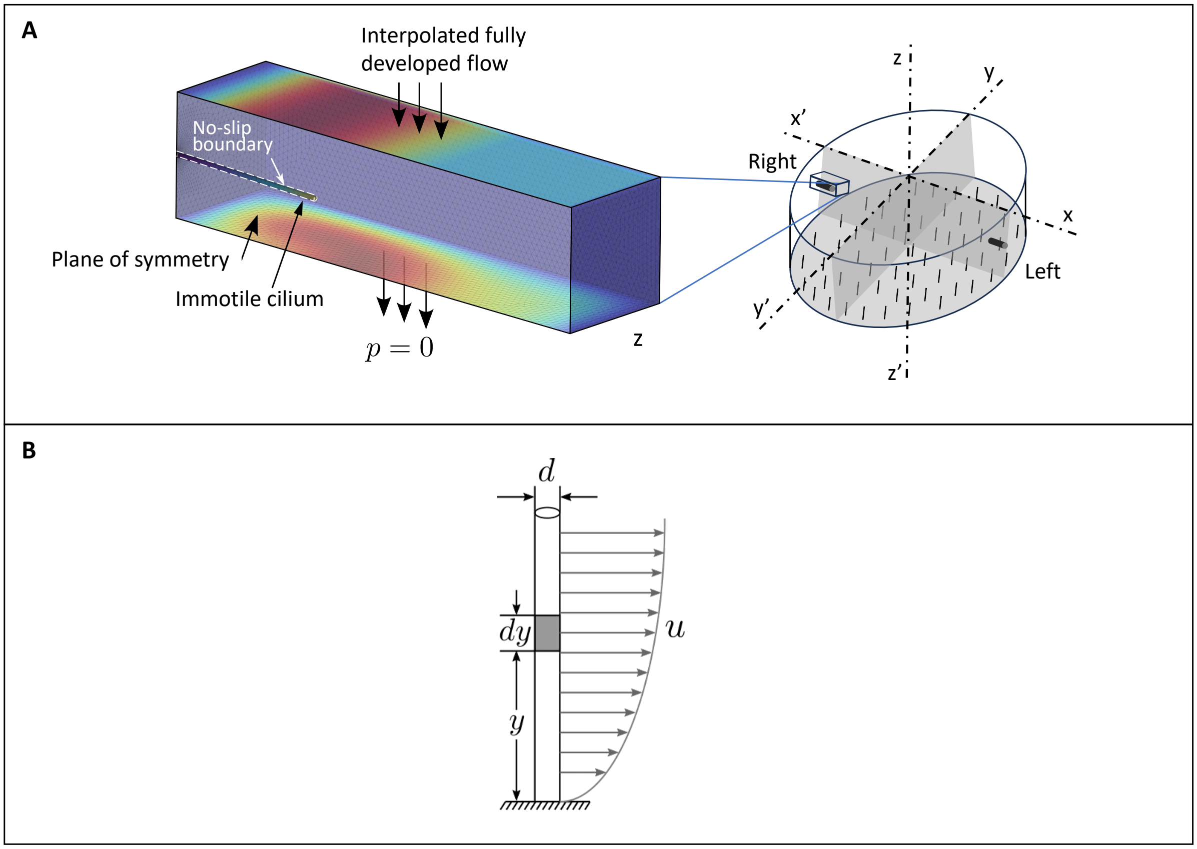

Fig. S17: (A) COMSOL model setup (left side) for computing the tip deflection of primary cilia within a control volume surrounding the primary cilium. Schematic (right side) illustrating the location of immotile cilia on the left and right side of the node along the vertical plane xz. (B) Schematic of a primary cilium subjected to viscous fluid loading: the cilium of diameter d is modeled as a cantilever beam clamped at the base and free at the tip, with an infinitesimal element dy experiencing drag from a parabolic channel flow u.

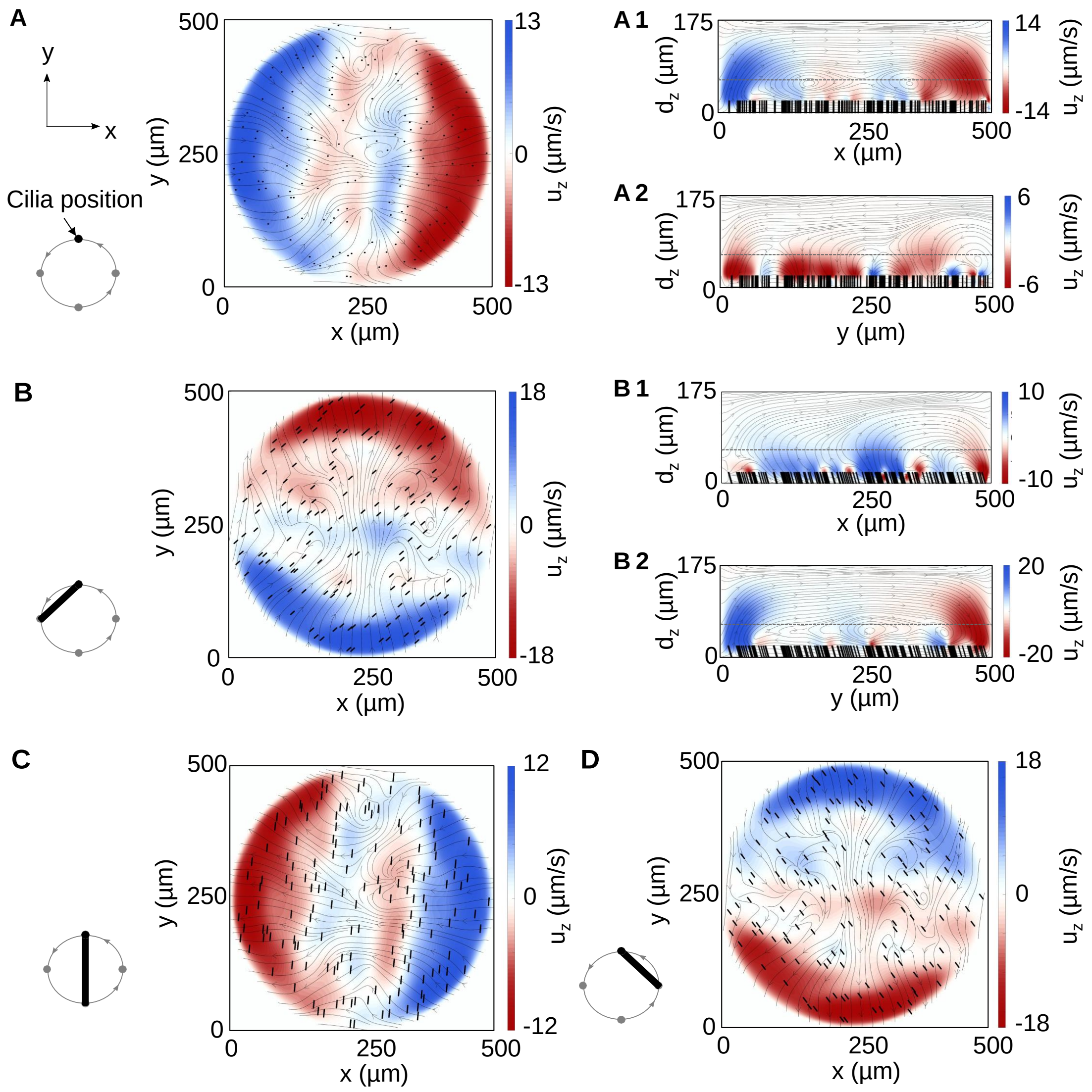

Fig. S18: Variation of the prominent velocity component (u_z) in the artificial node over a flow cycle at the onset of effective stroke 'A', at the middle of effective stroke 'B', at the end of effective or start of recovery stroke 'C', and at the end of recovery stroke 'D'. The first row displays the flow in the xy-plane (at $60\,\mu\text{m}$ above the node base), the second row shows the flow in the xz-plane (at $y = 250\,\mu\text{m}$, i.e., at the center), and the third row represents the flow in the yz-plane (at $x = 250\,\mu\text{m}$, at the center). Similar response is observed for the other two cases, i.e., (c & d). Schematics in each subfigure 'A-D' show the projected view of motile cilia position at the selected time instants. Colors represent the magnitude of u_z (scaled down), lines represent the flow streamlines, and the dashed line in the xz and yz-planes in 'A' and 'B' represents the position of immotile cilia. The direction of the u_z alternates between left-to-right and back-to-front (or vice versa) every quarter of a cycle. The peak values are observed closer to the periphery of the node. An animation of the flow field is shown in Movie S18.

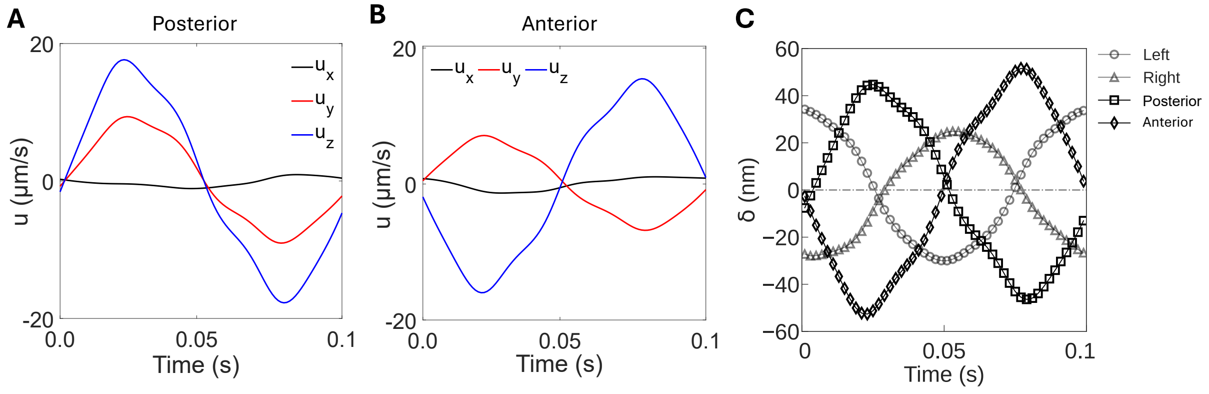

Fig. S19: (A-B) Variation of scaled-down peak velocity components experienced by the front and back primary cilia in the artificial embryonic node. The velocities again show the most significant velocity component is in the z-direction. Additionally, the posterior-anterior pairs are complementary, exhibiting a phase difference of $90°$ like around the left and right primary cilia. (C) Tip deflection of the primary cilia on the posterior and anterior side of the node with an average cilium length of $5\,\mu\text{m}$, diameter $200\,\text{nm}$ and a flexural rigidity of $4.5 \times 10^{-22}\ \text{Nm}^2$. The posterior cilium shows more deflection in both directions − upper/ventral and lower/dorsal side. Left and right cilia deflections from Fig. 4 are added here for the full comparison of immotile cilia deflection in the node.

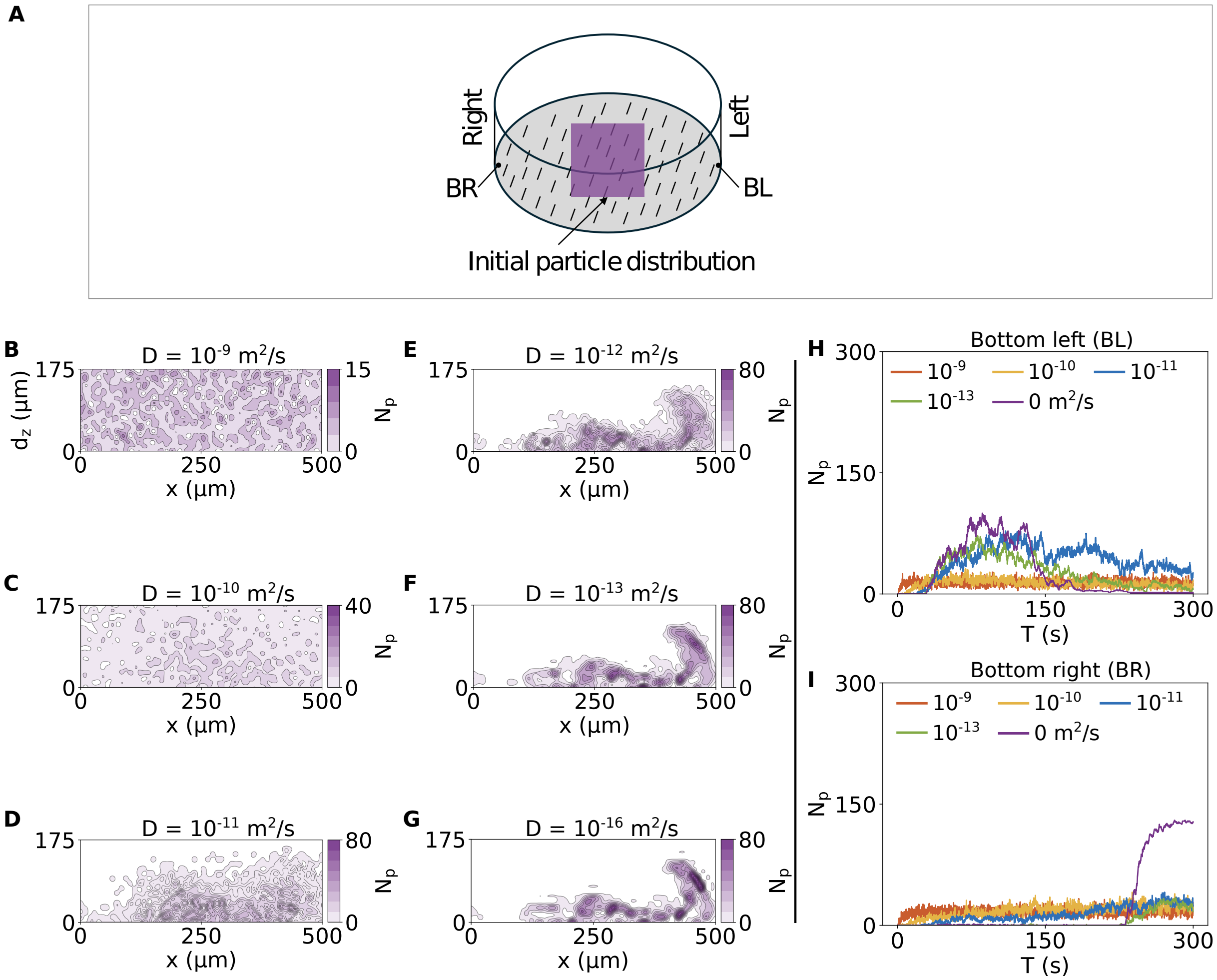

Fig. S20: (A) Schematic illustrating the initial position of particles spread over the central cross-section of the node and to a thickness of $20\,\mu\mathrm{m}$. The regions of interest where particles are tracked on the left and right side of the node include: bottom right (BR), and bottom left (BL). (B-G) Contour plots showing particle distribution at $50\,\mathrm{s}$ corresponding to different diffusion coefficients. (H-I) Time evolution of particle distribution at the bottom left and at the bottom right for various diffusion coefficients and for purely convective flow.

Table S1. SU-8 photolithography parameters

d (μm)	SU-8	Spin rpm ($30\ Sec$)	Bake (min)		UV dose (mJ/cm^2)	Post-bake (min)		Develop (min)
			$65\ ^0C$	$95\ ^0C$		$65\ ^0C$	$95\ ^0C$	
175	2075	1250	7	35	300	5	13	16+16
90	2050	1800	5	12	225	3	9	10+10
50	2050	3500	3	7	160	1	6	6+6

Movie S1. Cilia with a radius of $r = 1\ \mu\mathrm{m}$ and length $L \approx 23\ \mu\mathrm{m}$ are actuated to perform a tilted conical motion (TCM) with $(\theta = \psi \approx 25^0)$ using the two-magnet actuator. When observed from the top, the path traced by a cilium tip is shown to pass through the base of the cilium indicating a typical TCM.

Movie S2. A fast loopy-drift motion is represented by a spiral track around a cilium where the particle is trapped within the cilium vortex and the outer spiral track represents a slower radial-drift motion (corresponding to Fig. 2(B)). The fluid flows at a much higher velocity within the vortex than just outside it. The particles are traced in the artificial embryonic node at a depth of $d_z/L = 0.6$. The original grayscale movie is processed using ImageJ to identify the cilium and the particles as white spots readable by the software for particle tracking.

Movie S3. Loopy motion of particles/ fluid in all the six layers with $d_z/L = 0.6 - 7.1$ (corresponding to Fig. S6) captured with a high frame rate of 60 fps and played at a rate of 10 fps to better appreciate the loopy motion. Since all the particles move in phase, the loopy motion is synchronized throughout the node. Nodal left and right in all the movies are inverted.

Movie S4. Streamlined particle motion representing the steady-state fluid flow in the node with $w/d = 3$ (corresponding to Fig. 2(C)) obtained by setting the movie frame-rate equal to cilia actuation frequency of 10 Hz. Synchronizing the movie frame rate with (ω) makes the cilia appear still. The movie shows high-velocity circulatory fluid motion within the cilia vortices that extend up to $(d_z/L = 1.9)$. Some of the particles move at an average velocity (streamlined) of around $35\mu/s$ around the cilia undergoing TCM at $\omega = 10$ Hz. The generated nodal flow shown at a speed of $1x$ (i.e. real time) in different measurements planes, gives the true perspective of low nodal-flow velocities.

Movie S5. Nodal flow in the circular artificial embryonic node with $w/d = 3$ (corresponding to Fig. 2), in which the cilia are actuated at $\omega = 10\,\mathrm{Hz}$. In the beginning of the movie, cilia undergoing TCM at 1 Hz are shown. In the later part of the movie, the speed is increased to 10x to more easily appreciate the characteristics of the nodal flow generated. The leftward flow in the bottom part and rightward in top part generates a circulatory flow in the xz-plane which can be clearly appreciated at 10x speed in the layers traced at $d_z = 105 - 165\ \mu\mathrm{m}$ where the particles in focus flow rightwards and the out-of-focus particles below move leftwards. To further enhance the flow visualization, the frames in the traced layers are converted to gray-scale images using Fiji/ImageJ and the particle tracks (red lines), generated through a plugin TrackMate, are superimposed on the movie frames in the last part of the movie.

Movie S6. Square artificial embryonic node with $w/d = 3$ (corresponding to Fig. S8) showing a typical flow similar to the circular nodes with $w/d = 3$. The movie first shows the cilia TCM, followed by the fluid motion at different depths, and finally the particle tracks are superimposed.

Movie S7. Nodal flow in the triangular artificial embryonic node with $w/d = 3$ shown in Fig S9, showing first the cilia TCM, followed by the fluid motion at different depths, and finally the particle tracks are superimposed.

Movie S8. Nodal flow in the triangular artificial embryonic node with $w/d = 3$ shown in Fig S10. The cilia exhibit a weaker TCM than for the other results shown, with $\theta = 36.5^0$ and $\psi = 13.5^0$. The movie first shows the cilia TCM, followed by the fluid motion at different depths, and finally the particle tracks are superimposed.

Movie S9. Nodal flow in a triangular artificial embryonic node with $w/d = 3$ at two actuation frequencies. Identical rightward directional flow produced in the uppermost part of the artificial embryonic node at $d_z = 165$ μm at two different cilia beat frequencies of 5 Hz and 50 Hz shows the conserved nature of the nodal flow. Movie speeds are selected to better appreciate the identical flow produced at two different cilia beat frequencies. Movie corresponds to Fig. S9 and Fig. S11

Movie S10. Nodal flow in a circular artificial embryonic node with $w/d = 5.5$, corresponding to Fig. S13(A-E). The flow is multidirectional throughout the node, with a net leftward flow in the lower part and a predominantly rightward flow in the upper part. The movie first shows the cilia TCM (at $\omega = 1$ Hz), followed by the fluid motion at different depths $\omega = 10$ Hz, and finally the particle tracks are superimposed.

Movie S11. Nodal flow in a circular artificial node with $w/d = 10$, corresponding to Fig. S13(F-H). The flow is multidirectional throughout the node. The movie first shows the cilia TCM (at $\omega = 1$ Hz), followed by the fluid motion at different depths $\omega = 10$ Hz, and finally the particle tracks are superimposed.

Movie S12. Square artificial embryonic node with $w/d = 5.5$ (corresponding to Fig. S14(A-E)) showing a typical flow also observed in the circular node with $w/d = 5.5$. The movie first shows the cilia TCM, followed by the fluid motion at different depths, and finally the particle tracks are superimposed.

Movie S13. Shallow Square artificial embryonic node with $w/d = 10$ ((corresponding

to Fig. S14(F-H)) showing a typical flow also observed in the shallow circular node with $w/d = 10$. The movie first shows the cilia TCM, followed by the fluid motion at different depths, and finally the particle tracks are superimposed.

Movie S14. Triangular artificial embryonic node with $w/d = 5.5$ (corresponding to Fig. S15(A-E)) showing a typical flow also observed in the circular and square nodes with $w/d = 5.5$.The movie first shows the cilia TCM, followed by the fluid motion at different depths, and finally the particle tracks are superimposed.

Movie S15. Shallow triangular artificial embryonic node with $w/d = 10$ ((corresponding to Fig. S15(F-H)) showing a typical flow also observed in the circular and square nodes with $w/d = 10$. The movie first shows the cilia TCM, followed by the fluid motion at different depths, and finally the particle tracks are superimposed.

Movie S16. Top and side views of cilia, modeled as rigid bodies undergoing TCM motion about their bases, are used in a computational model to simulate the nodal flow. The cilia distribution and node geometry mimic the artificial embryonic node shown in Fig. 2.

Movie S17. Variation of cross-sectional flow in the artificial embryonic node, extracted from the computational model, over a complete rotation of the cilia undergoing TCM. The schematic diagram in the upper center shows the positions and orientation of the cross-sectional plane considered for flow visualization within the node.

Movie S18. Variation of the prominent z-component of velocity in the bottom part of the node at $d_z/L = 2.6$, and across the cross sections of the artificial embryonic node. The

schematic diagram in the upper center shows the positions and orientations of the cross-sectional planes considered for flow visualization within the node.

Movie S19. Maximum deflection of immotile cilia on the left and right sides of the node under the application of the scaled-down prominent z-component flow velocity, using a COMSOL model. The cilia have a maximum length of $7\ \mu\mathrm{m}$ and a minimum flexural rigidity of $0.7 \times 10^{-22}\ \mathrm{Nm}^2$. The left-side cilium exhibits larger deflections in both the dorsal and ventral directions compared to the right-side cilium.

Movie S20. Transportation of particles from the source located at the bottom central portion of the node under a purely convective behavior for a time period of $300s$, which corresponds to 3000 cilia beat cycles undergoing TCM at $10\ \mathrm{Hz}$. The side and top views of the node show particle movement within the node, giving a three-dimensional perspective of the particle distribution.

REFERENCES

1. H. Hamada, C. Meno, D. Watanabe, Y. Saijoh, Establishment of vertebrate left–right asymmetry. *Nat. Rev. Genet.* **3**, 103–113 (2002).

2. Á. Raya, J. C. I. Belmonte, Left–right asymmetry in the vertebrate embryo: From early information to higher-level integration. *Nat. Rev. Genet.* **7**, 283–293 (2006).

3. S. Nonaka, Y. Tanaka, Y. Okada, S. Takeda, A. Harada, Y. Kanai, M. Kido, N. Hirokawa, Randomization of left–right asymmetry due to loss of nodal cilia generating leftward flow of extraembryonic fluid in mice lacking KIF3B motor protein. *Cell* **95**, 829–837 (1998).

4. N. Hirokawa, Y. Okada, Y. Tanaka, Fluid dynamic mechanism responsible for breaking the left-right symmetry of the human body: The nodal flow. *Annu. Rev. Fluid Mech.* **41**, 53–72 (2009).

5. Y. Okada, S. Takeda, Y. Tanaka, J.-C. I. Belmonte, N. Hirokawa, Mechanism of nodal flow: A conserved symmetry breaking event in left-right axis determination. *Cell* **121**, 633–644 (2005).

6. K. Shinohara, A. Kawasumi, A. Takamatsu, S. Yoshiba, Y. Botilde, N. Motoyama, W. Reith, B. Durand, H. Shiratori, H. Hamada, Two rotating cilia in the node cavity are sufficient to break left–right symmetry in the mouse embryo. *Nat. Commun.* **3**, 622 (2012).

7. T. Nakamura, D. Saito, A. Kawasumi, K. Shinohara, Y. Asai, K. Takaoka, F. Dong, A. Takamatsu, J. A. Belo, A. Mochizuki, H. Hamada, Fluid flow and interlinked feedback loops establish left–right asymmetric decay of Cerl2 mRNA. *Nat. Commun.* **3**, 1322 (2012).

8. K. Minegishi, B. Rothé, K. R. Komatsu, H. Ono, Y. Ikawa, H. Nishimura, T. A. Katoh, E. Kajikawa, X. Sai, E. Miyashita, K. Takaoka, K. Bando, H. Kiyonari, T. Yamamoto, H. Saito, D. B. Constam, H. Hamada, Fluid flow-induced left-right asymmetric decay of Dand5 mRNA in the mouse embryo requires a Bicc1-Ccr4 RNA degradation complex. *Nat. Commun.* **12**, 4071 (2021).

9. X. Zhou, H. Sasaki, L. Lowe, B. L. Hogan, M. R. Kuehn, Nodal is a novel TGF-β-like gene expressed in the mouse node during gastrulation. *Nature* **361**, 543–547 (1993).

10. C. Meno, Y. Ito, Y. Saijoh, Y. Matsuda, K. Tashiro, S. Kuhara, H. Hamada, Two closely-related left-right asymmetrically expressed genes, lefty-1 and lefty-2: Their distinct expression domains, chromosomal linkage and direct neuralizing activity in *Xenopus* embryos. *Genes Cells* **2**, 513–524 (1997).

11. A. K. Ryan, B. Blumberg, C. Rodriguez-Esteban, S. Yonei-Tamura, K. Tamura, T. Tsukui, J. De La Peña, W. Sabbagh, J. Greenwald, S. Choe, D. P. Norris, E. J. Robertson, R. M. Evans, M. G. Rosenfeld, J. C. I. Belmonte, Pitx2 determines left–right asymmetry of internal organs in vertebrates. *Nature* **394**, 545–551 (1998).

12. H. Yoshioka, C. Meno, K. Koshiba, M. Sugihara, H. Itoh, Y. Ishimaru, T. Inoue, H. Ohuchi, E. V. Semina, J. C. Murray, H. Hamada, S. Noji, Pitx2, a bicoid-type homeobox gene, is involved in a lefty-signaling pathway in determination of left-right asymmetry. *Cell* **94**, 299–305 (1998).

13. P. Pennekamp, T. Menchen, B. Dworniczak, H. Hamada, Situs inversus and ciliary abnormalities: 20 years later, what is the connection? *Cilia* **4**, 1 (2015).

14. D. Bellomo, A. Lander, I. Harragan, N. A. Brown, Cell proliferation in mammalian gastrulation: The ventral node and notochord are relatively quiescent. *Dev. Dyn.* **205**, 471–485 (1996).

15. J. H. Cartwright, O. Piro, I. Tuval, Fluid-dynamical basis of the embryonic development of left-right asymmetry in vertebrates. *Proc. Natl. Acad. Sci. U.S.A.* **101**, 7234–7239 (2004).

16. Y. Okada, S. Nonaka, Y. Tanaka, Y. Saijoh, H. Hamada, N. Hirokawa, Abnormal nodal flow precedes situs inversus in iv and inv mice. *Mol. Cell* **4**, 459–468 (1999).

17. J. McGrath, M. Brueckner, Cilia are at the heart of vertebrate left–right asymmetry. *Curr. Opin. Genet. Dev.* **13**, 385–392 (2003).

18. Y. Tanaka, Y. Okada, N. Hirokawa, FGF-induced vesicular release of Sonic hedgehog and retinoic acid in leftward nodal flow is critical for left–right determination. *Nature* **435**, 172–177 (2005).

19. J. McGrath, S. Somlo, S. Makova, X. Tian, M. Brueckner, Two populations of node monocilia initiate left-right asymmetry in the mouse. *Cell* **114**, 61–73 (2003).

20. C. J. Tabin, K. J. Vogan, A two-cilia model for vertebrate left-right axis specification. *Genes Dev.* **17**, 1–6 (2003).

21. D. Smith, E. Gaffney, J. Blake, Discrete cilia modelling with singularity distributions: Application to the embryonic node and the airway surface liquid. *Bull. Math. Biol.* **69**, 1477–1510 (2007).

22. D. J. Smith, A. A. Smith, J. R. Blake, Mathematical embryology: The fluid mechanics of nodal cilia. *J. Eng. Math.* **70**, 255–279 (2011).

23. T. D. Montenegro-Johnson, A. A. Smith, D. J. Smith, D. Loghin, J. R. Blake, Modelling the fluid mechanics of cilia and flagella in reproduction and development. *Eur. Phys. J. E* **35**, 111 (2012).

24. J. H. Cartwright, O. Piro, I. Tuval, Chemosensing versus mechanosensing in nodal and Kupffer's vesicle cilia and in other left–right organizer organs. *Philos. Trans. R. Soc. B* **375**, 20190566 (2020).

25. S. Nonaka, H. Shiratori, Y. Saijoh, H. Hamada, Determination of left–right patterning of the mouse embryo by artificial nodal flow. *Nature* **418**, 96–99 (2002).

26. Y. Tanaka, A. Morozumi, N. Hirokawa, Nodal flow transfers polycystin to determine mouse left-right asymmetry. *Dev. Cell* **58**, 1447–1461.e6 (2023).

27. M. Delling, A. Indzhykulian, X. Liu, Y. Li, T. Xie, D. Corey, D. Clapham, Primary cilia are not calcium-responsive mechanosensors. *Nature* **531**, 656–660 (2016).

28. K. Mizuno, K. Shiozawa, T. A. Katoh, K. Minegishi, T. Ide, Y. Ikawa, H. Nishimura, K. Takaoka, T. Itabashi, A. H. Iwane, J. Nakai, H. Shiratori, H. Hamada, Role of Ca^{2+} transients at the node of the mouse embryo in breaking of left-right symmetry. *Sci. Adv.* **6**, eaba1195 (2020).

29. T. A. Katoh, T. Omori, K. Mizuno, X. Sai, K. Minegishi, Y. Ikawa, H. Nishimura, T. Itabashi, E. Kajikawa, S. Hiver, A. H. Iwane, T. Ishikawa, Y. Okada, T. Nishizaka, H. Hamada, Immotile cilia mechanically sense the direction of fluid flow for left-right determination. *Science* **379**, 66–71 (2023).

30. T. A. Katoh, T. Omori, T. Ishikawa, Y. Okada, H. Hamada, Biophysical analysis of mechanical signals in immotile cilia of mouse embryonic nodes using advanced microscopic techniques. *Bio Protoc.* **13**, e4715 (2023).

31. L. Djenoune, M. Mahamdeh, T. V. Truong, C. T. Nguyen, S. E. Fraser, M. Brueckner, J. Howard, S. Yuan, Cilia function as calcium-mediated mechanosensors that instruct leftright asymmetry. *Science* **379**, 71–78 (2023).

32. T. A. Katoh, T. Lange, Y. Nakajima, K. Yashiro, Y. Okada, H. Hamada, BMP4 regulates asymmetric Pkd2 distribution in mouse nodal immotile cilia and ciliary mechanosensing required for left–right determination. *Dev. Dyn.* **254**, 965–978 (2024).

33. T. A. Katoh, Function of nodal cilia in left-right determination: Mechanical regulation in initiation of symmetry breaking. *Biophys. Physicobiol.* **21**, e210018 (2024).

34. A. Shields, B. Fiser, B. Evans, M. Falvo, S. Washburn, R. Superfine, Biomimetic cilia arrays generate simultaneous pumping and mixing regimes. *Proc. Natl. Acad. Sci. U.S.A.* **107**, 15670–15675 (2010).

35. T. Ul Islam, Y. Bellouard, J. M. den Toonder, Highly motile nanoscale magnetic artificial cilia. *Proc. Natl. Acad. Sci. U.S.A.* **118**, e2104930118 (2021).

36. B. B. Venkataramanachar, J. Li, T. U. Islam, Y. Wang, J. den Toonder, Nanomagnetic elastomers for realizing highly responsive micro- and nanosystems. *Nano Lett.* **23**, 9203–9211 (2023).

37. D. J. Smith, J. R. Blake, E. A. Gaffney, Fluid mechanics of nodal flow due to embryonic primary cilia. *J. R. Soc. Interface* **5**, 567–573 (2008).

38. S. Khaderi, P. Onck, Fluid–structure interaction of three-dimensional magnetic artificial cilia. *J. Fluid Mech.* **708**, 303–328 (2012).

39. R. Zhang, J. den Toonder, P. R. Onck, Transport and mixing by metachronal waves in nonreciprocal soft robotic pneumatic artificial cilia at low Reynolds numbers. *Phys. Fluids* **33**, 092009 (2021).

40. J. H. Cartwright, N. Piro, O. Piro, I. Tuval, Embryonic nodal flow and the dynamics of nodal vesicular parcels. *J. R. Soc. Interface* **4**, 49–55 (2007).

41. M. T. Gallagher, T. D. Montenegro-Johnson, D. J. Smith, Simulations of particle tracking in the oligociliated mouse node and implications for left–right symmetry-breaking mechanics. *Philos. Trans. R. Soc. B* **375**, 20190161 (2020).

42. N. Hirokawa, Y. Tanaka, Y. Okada, S. Takeda, Nodal flow and the generation of left-right asymmetry. *Cell* **125**, 33–45 (2006).

43. S. Yoshiba, H. Shiratori, I. Y. Kuo, A. Kawasumi, K. Shinohara, S. Nonaka, Y. Asai, G. Sasaki, J. A. Belo, H. Sasaki, J. Nakai, B. Dworniczak, B. E. Ehrlich, P. Pennekamp, H. Hamada, Cilia at the node of mouse embryos sense fluid flow for left-right determination via Pkd2. *Science* **338**, 226–231 (2012).

44. B. A. Evans, B. L. Fiser, W. J. Prins, D. J. Rapp, A. R. Shields, D. R. Glass, R. Superfine, A highly tunable silicone-based magnetic elastomer with nanoscale homogeneity. *J. Magn. Magn. Mater.* **324**, 501–507 (2012).

45. J. R. Blake, A note on the image system for a stokeslet in a no-slip boundary. *Math. Proc. Camb. Philos. Soc.* **70**, 303–310 (1971).

46. D. Ershov, M.-S. Phan, J. W. Pylvänäinen, S. U. Rigaud, L. Le Blanc, A. Charles-Orszag, J. R. Conway, R. F. Laine, N. H. Roy, D. Bonazzi, G. Duménil, G. Jacquemet, J.-Y. Tinevez, TrackMate 7: Integrating state-of-the-art segmentation algorithms into tracking pipelines. *Nat. Methods* **19**, 829–832 (2022).
